# BAP1 Loss Is Associated with Higher ASS1 Expression in Epithelioid Mesothelioma: Implications for Therapeutic Stratification

**DOI:** 10.1158/1541-7786.MCR-22-0635

**Published:** 2023-01-20

**Authors:** Sarah E. Barnett, Jenna Kenyani, Martina Tripari, Zohra Butt, Rudi Grosman, Francesca Querques, Liam Shaw, Luisa C. Silva, Zoe Goate, Stefan J. Marciniak, Doris M. Rassl, Richard Jackson, Lu-Yun Lian, Peter W. Szlosarek, Joseph J. Sacco, Judy M. Coulson

**Affiliations:** 1 Molecular Physiology and Cell Signalling, University of Liverpool, Liverpool, United Kingdom.; 2 Biochemistry and Systems Biology, University of Liverpool, Liverpool, United Kingdom.; 3 Cambridge Institute for Medical Research, Cambridge, United Kingdom.; 4 Papworth Hospital NHS Foundation Trust, Cambridge, United Kingdom.; 5 Molecular and Clinical Cancer Medicine, Institute of Systems, Molecular and Integrative Biology, University of Liverpool, Liverpool, United Kingdom.; 6 Liverpool Clinical Trials Centre, University of Liverpool, Liverpool, United Kingdom.; 7 Barts Cancer Institute, Queen Mary University of London, London, United Kingdom.; 8 Clatterbridge Cancer Centre NHS Foundation Trust, Wirral, United Kingdom.

## Abstract

The nuclear deubiquitylase BRCA1-associated protein 1 (BAP1) is frequently inactivated in malignant pleural mesothelioma (MPM) and germline *BAP1* mutation predisposes to cancers including MPM. To explore the influence on cell physiology and drug sensitivity, we sequentially edited a predisposition mutation (*w-*) and a promoter trap (*KO*) into human mesothelial cells. *BAP1^w-/KO^* MeT5A cells express less BAP1 protein and phenocopy key aspects of BAP1 loss in MPM. Stable isotope labeling with amino acids in cell culture–mass spectrometry revealed evidence of metabolic adaptation, with concomitant alteration of cellular metabolites. In MeT5A, BAP1 deficiency reduces glycolytic enzyme levels but increases enzymes involved in the tricarboxylic acid cycle and anaplerotic pathways. Notably both argininosuccinate synthase 1 (ASS1), essential for cellular synthesis of arginine, and its substrate aspartate, are elevated in *BAP1^w-/KO^* MeT5A cells. Likewise, ASS1 expression is higher in BAP1-altered MPM cell lines, and inversely correlates with BAP1 in The Cancer Genome Atlas MESO dataset. Elevated ASS1 is also evident by IHC staining in epithelioid MPM lacking nuclear BAP1 expression, with improved survival among patients with BAP1-negative/ASS1-expressing tumors. Alterations in arginine metabolism may sensitize cells to metabolic drugs and we find that BAP1-negative/ASS1-expressing MPM cell lines are more sensitive to ASS1 inhibition, although not to inhibition of purine synthesis by mizoribine. Importantly, *BAP1^w-/KO^* MeT5A become desensitized to arginine deprivation by pegylated arginine deiminase (ADI-PEG20), phenocopying BAP1-negative/ASS1-expressing MPM cell lines.

**Implications:**

Our data reveal an interrelationship between BAP1 and arginine metabolism, providing a potential means of identifying patients with epithelioid MPM likely to benefit from ADI-PEG20.

## Visual Overview

**Figure:**
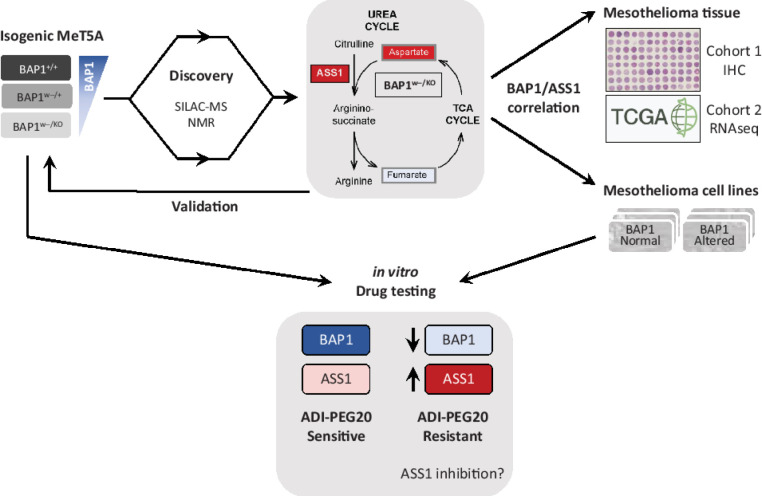

## Introduction

Malignant pleural mesothelioma (MPM) originates in the mesothelial lining of the pleural cavity and is strongly associated with asbestos exposure several decades prior to diagnosis. It remains a disease of significant unmet need with high global mortality rates and rising incidence, despite asbestos bans in many countries (1). Exposure to similarly sized carbon nanotubes also initiates MPM in mice, raising future concerns (2). Histologically, MPM are classified as epithelioid, sarcomatoid, and biphasic, which encompass a continuous spectrum of disease (3). Treatment with cisplatin and pemetrexed, with or without the VEGF antibody bevacizumab (4), results in only modest improvement in survival. Recent randomized trials have shown survival benefit for both immune checkpoint inhibitors (5) and arginine deprivation (6, 7), offering new promise for patients with MPM. However, benefits vary widely with histologic and genetic features in this heterogeneous disease. More complete understanding of MPM biology may help broaden the options available and allow selection of the most appropriate therapies for individual patients.

MPM has few actionable oncogenic driver mutations but is instead characterized by tumor suppressor inactivation. One common example is BRCA1-associated protein 1 (*BAP1)*, an early truncal change that may occur with or without inactivation of neurofibromin-2 (*NF2)* and/or cyclin-dependent kinase inhibitor 2A/2B *(CDKN2A/B*; refs. 8–10). *BAP1* mutation typically results in catalytic inactivation or loss of BAP1 nuclear localization. It is predominantly associated with epithelioid MPM, but also occurs in biphasic and occasionally sarcomatoid subtypes. Although the reported frequency for somatic *BAP1* loss-of-function mutation varies, IHC studies show loss of nuclear BAP1 (nBAP1) in up to 60% of all patients with MPM and 80% of epithelioid cases (11–14). Germline *BAP1* mutation underpins a cancer predisposition syndrome where family members develop multiple cancers, most commonly MPM, peritoneal mesothelioma, and uveal melanoma (13, 15).

Despite initial identification as a tumor suppressor in lung and breast cancer (16), somatic *BAP1* mutation is infrequent in epithelial cancers. Indeed, CRISPR/Cas9 screens show *BAP1* is an essential/fitness gene (17), suggesting cancers may need to reprogram essential pathways to tolerate BAP1 loss of function. One explanation for the limited palette of tumors promoted by BAP1 loss is that it activates a specific apoptotic program in other cell backgrounds (18). Indeed, roles for BAP1 appear to be highly complex and context dependent. For example, BAP1 expression can promote pro-oncogenic pathways, including breast cancer cell proliferation and radioresistance in head and neck cancer (19, 20). BAP1 depletion has also been shown to reduce cell proliferation through S-phase accumulation in MPM (14), while it is suggested to function primarily as a metastasis suppressor in uveal melanoma (21). Clinically, systematic analyses show that although BAP1 loss of function is associated with worse prognosis in uveal melanoma and renal clear cell carcinoma (RCCC), it predicts better prognosis in patients with MPM (22).

Despite these complexities, genetically engineered mouse models (GEMM) provide fundamental evidence that BAP1 is tumor suppressor. Mice with heterozygous germline *Bap1* mutation spontaneously develop tumors of the ovary, lung, or breast and, in response to asbestos exposure, develop MPM more frequently and rapidly than wild-type mice (23–25). More recently, deletion of *Bap1* in the mesothelial lining of the thoracic cavity was shown to require *Nf2* and *Cdkn2ab* inactivation to develop tumors that recapitulate key histologic, inflammatory, and gene expression features of human MPM (26). However, the critical roles of BAP1 as a tumor suppressor in MPM remain incompletely understood, and utilization of BAP1 status for therapeutic stratification has not yet been realized. In addition to GEMMs, modification of human cell lines by reexpression or genetic deletion of BAP1 can provide useful insights, although to date cancer cell lines with heterogenous genetic alterations have been employed (27–29).

At a molecular level, *BAP1* encodes a nuclear deubiquitylase that regulates processes including the cell cycle, DNA damage repair, and metabolism, through fundamental roles in protein stabilization, histone modification, and transcriptional regulation (13, 30), suggesting potential targets for therapeutic intervention in MPM with BAP1 loss. Recent clinical studies investigating enhancer of zeste 2 polycomb repressive complex 2 subunit (EZH2) and PARP inhibitors suggest some clinical efficacy for both (7, 31). However, the tazemetostat study only recruited BAP1-negative patients and rucaparib efficacy did not relate to BAP1 status. Taken together with the relatively low efficacy of tazemetostat, this highlights the need for further study into understanding and targeting BAP1 dependencies.

Here we functionally interrogated cellular adaptation to BAP1 deficiency, to define the consequences for cell physiology and potential therapy. To provide a clean model for acquisition of BAP1 alterations, we generated isogenic mesothelial cells with sequential introduction of clinically relevant *BAP1* mutations. These *BAP1^w-/KO^* cells express sufficient low-level BAP1 to support viability, but effectively mimic *BAP1*-mutated MPM. We employed proteomic and metabolomic analyses to profile BAP1 dependencies, which included metabolic reprogramming. Notably, upregulation of argininosuccinate 1 synthase (ASS1), an enzyme essential for cellular synthesis of arginine and its downstream metabolites, was identified in *BAP1^w-/KO^* cells and in BAP1-deficient MPM cells or tumor samples. Importantly, we found that BAP1 status is associated with sensitivity to metabolic drugs including pegylated arginine deiminase (ADI-PEG20), and thus may be exploited in patient stratification for relevant therapeutic interventions such as arginine deprivation.

## Materials and Methods

### Cell culture

MeT5A cells (ATCC, obtained 2012, RRID:SCR_003193) were cultured in Media 199 (1 g/L glucose) supplemented as described previously (32) with selection antibiotics as appropriate. MPM cell lines (for RRID, see Supplementary Table S1) were sourced from ATCC (NCI or MSTO, obtained 2012/2104) or Mesobank (33) [MESO (34) obtained 2017, and MPM# (35) obtained 2015; www.mesobank.com]. MPM cells were cultured in RPMI supplemented with 10% FBS (2 g/L glucose), and for MESO cell lines 20 ng/mL EGF (Peprotech), 1 μg/mL hydrocortisone (Sigma-Aldrich), and 2 μg/mL heparin (Sigma-Aldrich). All cells were grown in a humidified incubator at 37°C and 5% CO_2_. Cells were routinely verified as *Mycoplasma* free during culture, using EZ-PCR *Mycoplasma* (Geneflow) or e-Myco Plus PCR (Bulldog bio) detection kits. All cells were authenticated by short tandem repeat (STR) profiling using the GenePrint10 system (Promega). This was last performed in September 2018; all lines achieved a DSMZ score >0.8 (Supplementary Table S1) and were batch frozen, with all experiments performed between passage 3 and 20 after thawing the STR-profiled cells.

### Genome editing of *BAP1*

The *BAP1* locus was edited using recombinant adeno-associated virus (rAAV) homologous recombination strategies designed by Horizon Discovery to sequentially target alleles with a predisposition point mutation (w-) and a promoter trap (KO). HEK293T cells (RRID:SCR_003193) were cotransfected with the genome-editing cassette and AAV-2 helper plasmid (Plasmid Factory) using Lipofectamine LTX (Invitrogen) and rAAVs extracted 48 hours later using Virakit (Virapur); the viral titre was estimated by real-time PCR. To establish the MeT5A-*BAP1^w-/+^* line, clonal MeT5A-*BAP1^+/+^*(C2) cells were infected with purified rAAV-0365 for 72 hours, then seeded into 96-well plates at 10–1,000 cells/well in selection media (0.7 μg/mL puromycin, Sigma-Aldrich). To establish MeT5A-*BAP1^w-/KO^* cells, the MeT5A-*BAP1^w-/+^* line was infected with rAAV-0612 and selected in 0.7 μg/mL puromycin and 0.1 mg/mL G418 (ForMedia). After 2 weeks, colonies were lysed in Direct PCR Lysis Reagent (Viagen Biotech) and screened with primers that distinguished the edited locus using GoTaq Flexi (Promega). Positive colonies were single cell diluted and rescreened to establish clones, which were sequence verified (Dundee Sequencing Services) and authenticated by STR profiling.

### Protein extraction and immunoblotting

Whole-cell extracts were prepared by hot lysis in Laemmli buffer, with protein concentrations determined and immunoblotting performed as described previously (36). Primary antibodies were mouse anti-ASS1 (2C10 MABN704, Sigma-Aldrich; RRID:AB_2927674), BAP1 (C-4 sc-28383, Santa Cruz Biotechnology; RRID:AB_626723), PGK1 (22C5D8 ab113687, Abcam; RRID:AB_10861977), and β-actin (ab6276, Abcam; RRID:AB_2223210); rabbit anti-AK4 (HPA049461, Sigma-Aldrich; RRID:AB_2680776), DHFR (ab124814, Abcam; RRID:AB_10975115), ENO2 (#9536, Cell Signaling Technology; RRID:AB_2099308), GLS (ab156876, Abcam; RRID:AB_2721038), histone H2A (07-146, Upstate; RRID:AB_310394), H2A-Ub (#8240, Cell Signaling Technology; RRID:AB_10891618), MDH2 (HPA019716, Sigma-Aldrich; RRID:AB_1853680), OGT (#5368S, Cell Signaling Technology; RRID:AB_11217621), PGAM1 (ab96622, Abcam; RRID:AB_10687155), PGM2 (ab151746, Abcam; RRID:AB_2927675), PYGL (HPA004119, Sigma-Aldrich; RRID:AB_1079723), RRM1 (3388, Cell Signaling Technology; RRID:AB_2180382) and β-actin (A2066, Sigma-Aldrich; RRID:AB_476693), or goat anti-ALDOC (D-14 sc-12066, Santa Cruz Biotechnology; RRID:AB_2226594), IDH3B (ab118287, Abcam; RRID:AB_10899317), and SUCLG2 (A-15 sc-99646, Santa Cruz Biotechnology; RRID:AB_10843272). Proteins were visualized using secondary antibodies conjugated to IRDyes and the LI-COR Odyssey 2.1 system; 16-bit images were quantified using ImageStudioLite (RRID:SCR_013715).

### Cell proliferation and therapeutic efficacy assays

MeT5A cells were seeded at suitable density and monitored for up to 5 days. The relative cell number was determined by direct counting, cell growth by CellTiter-Glo assay (Promega), or cell confluence by live imaging using an IncuCyte S3 (Essen Bioscience; RRID:SCR_019874). For drug assays, cells were seeded in 96-well plates at appropriate densities for each cell line between 1 × 10^3^ and 4.5 × 10^3^ cells/well. After 24 hours, cells were treated with vehicle, α-methyl-DL-aspartic acid (αMDLA, Biosynth AG), mizoribine (Sigma-Aldrich), or ADI-PEG20 (Polaris Pharmaceuticals Inc) at the indicated doses, with viability (CellTiter-Glo) and/or confluence (IncuCyte) monitored for up to 4 days.

### Cell-cycle analysis

Exponentially growing cells were fixed prior to 7-aminoactinomycin D (Invitrogen) staining to determine DNA content. The fluorescence of single cells was quantified using a Attune NxT system and analyzed using FlowJo v10.8.1 software (RRID:SCR_008520).

### Mass spectrometry

For stable isotope labeling with amino acids in cell culture (SILAC), cells were maintained for 6 passages in arginine/lysine-free media with normal supplements and amino acids (Sigma-Aldrich) in light, medium, or heavy configurations (37) at final concentrations of 84 mg/L arginine, 146 mg/L lysine, and 200 mg/L proline. Labeling efficiency was >98% with <2% proline conversion. For in-gel digestion and peptide extraction, lysates from MeT5A-*BAP1^+/+^*(C2), *BAP1^w-/+^*, and *BAP1^w-/KO^* (C3.1 or C5.1) were mixed (1:1:1, 90 mg total) and separated by SDS-PAGE. Proteins were extracted as described previously (36). Briefly, gel pieces were de-stained with 50% acetonitrile/50% 100 mmol/L NH_4_HCO_3_, reduced with 10 mmol/L dithiothreitol and alkylated with 50 mmol/L iodoacetamide. Following acetonitrile dehydration and incubation with Trypsin Gold (Promega), peptides were extracted in acetonitrile, vacuum dried and resuspended in 1% formic acid. For filter-aided sample preparation (FASP), MESO-8T, MESO-12T, and MeT5A-*BAP1^+/+^*(C2) lysates were mixed (1:1:1, 300 mg total) and diluted in 8 mol/L urea in 50 mmol/L Tris and 75 mmol/L NaCl. Samples were centrifuged for 20 minutes at 8,000 × *g* in Amicon Ultra 0.5 mL 10 kDa molecular weight cut-off filters (Merck). Following two washes with 8 mol/L urea buffer and two with 50 mmol/L NH_4_HCO_3_, samples were reduced and alkylated with 10 mmol/L tris(2-carboxyethyl)phosphine and 40 mmol/L 2-chloroacetamide in 50 mmol/L NH_4_HCO_3_ for 30 minutes then washed twice with 50 mmol/L NH_4_HCO_3_. Samples were digested with Lys-C (0.5 mg/100 mg protein) for 2 hours at room temperature and Trypsin Gold (2 mg/100 mg protein) overnight at 37°C before peptides were eluted by centrifugation. Mass spectrometry (MS) analysis of the isogenic cell panel was performed in-house as described previously (36) with two replicate runs, or by the Proteomics Research Technology Platform (University of Warwick, Coventry, United Kingdom) for samples prepared via FASP. All MS RAW files were analyzed using MaxQuant (RRID:SCR_014485) equipped with the Andromeda search engine, and further processed using Microsoft Excel (RRID:SCR_016137) and Perseus (RRID:SCR_015753).

### Metabolomics

Pellets of MeT5A-*BAP1^+/+^*(C2) and *BAP1^w-/KO^* (C5.1) cells were collected in triplicate for three biological replicates. For metabolite extraction, cells were mixed with ice-cold 50% acetonitrile/water and subject to 3×30 seconds sonication (23 KHz, 10 μm amplitude) with 30-second intervals, then centrifuged for 10 minutes at 4°C and 12,000 × *g*. Supernatants were lyophilized for 16 hours and mixed with 190 μL buffer [100 mmol/L sodium phosphate (pH7.4), 0.1 mmol/L 3-(Trimethylsilyl)propionic-2,2,3,3-d_4_ acid sodium salt (Sigma-Aldrich), 1.2 mmol/L sodium azide (Sigma-Aldrich)]. Spectra were acquired in 3 mm nuclear magnetic resonance (NMR) tubes using a Bruker Avance III 700 MHz NMR spectrometer fitted with a 5 mm [^1^H, ^15^N, ^13^C]-TCI Cryoprobe and SampleJet. All ^1^H NMR spectra were acquired at 25°C (± 0.1°C) and processed (phase and baseline correction) using automation for consistency. CPMG and 1D NOE pulse sequences were used for acquisition, with a spectral width of 30 ppm, 48K complex points and 32 scans (1D NOE) or 256 scans (CPMG). Acquired spectra that passed quality checks were used in statistical analysis and metabolite identification using in-house standards library and the Chenomx NMR Suite (RRID:SCR_014682). For statistical analysis, each peak was individually bucketed. Prior to analysis each spectrum was centred to the median and samples were pareto scaled to each other. Principal component analysis (PCA) and *t* tests were performed.

### RNA extraction and qRT-PCR

Total RNA was extracted and qRT-PCR performed using SYBR Green supermix as described previously (36). Primer sequences were: *ACTB* (For: 5′-CACCTTCTACAATGAGCTGCGTGTG-3′, Rev: 5′-ATAGCACAGCCTGGATAGCAACGTAC-3′), and *ASS1* (For: 5′-GAAGTGCGCAAAATCAAACAAG-3′, Rev: 5′-GATGTACACCTGGCCCTTG). *ASS1* was normalized to *ACTB* and represented as 2^−[ΔΔ^^*C*_t_^^]^ relative to MeT5A*-BAP1^+/+^*.

### IHC staining and scoring

NHS Health Research Authority National Research Ethics Service approval, conforming to the principles of the Declaration of Helsinki and with written informed patient consent, was conferred by the supplying tissue banks. Antibodies were optimized on whole MPM sections from Royal Papworth Hospital Research Tissue bank (NHS REC 18/EE/0269). Tissue microarrays (TMA) containing 3- to 4-μm-thick and 800 μm diameter cores from MPM samples (4 cores/patient) were obtained from Mesobank (ref. 33; http://www.mesobank.com/; NHS REC 18/EE/0161). Antigen retrieval was performed using the PT Link Pretreatment module (Agilent Technologies). Slides were incubated at high-pH (pH9.0; BAP1, pan-cytokeratin) or low-pH (pH6.0; ASS1) at 96°C for 20 minutes. IHC was performed using the DAKO Envision FLEX Kit (Agilent). Slides were incubated for 30 minutes with mouse anti-pan-cytokeratin (1:80; MNF116/MA1-26237; Thermo Fisher Scientific; RRID:AB_794730), anti-BAP1 (1:400; C-4/sc-28383; Santa Cruz Biotechnology; RRID:AB_626723), or rabbit anti-ASS1 (1:200; HPA020934; Sigma-Aldrich; RRID:AB_1845118), and counterstained with hematoxylin. Tumor cell nuclear immunoreactivity for BAP1 (13) was scored by three independent observers blinded to clinical and/or pathologic data; non-MPM cells acted as internal positive controls. ASS1 stained slides were scanned (x40, .svs format) using a Leica Aperio CS2 for and QuPath Bioimage analysis v.0.2.0 (https://QuPath.github.io/; RRID:SCR_018257; ref. 38) used to generate tumor H-scores. There was some sample attrition as cores that were missing, had insufficient tumor tissue, were nonepithelioid or not scorable for BAP1, were excluded from analysis. Cell detection identified all cells within a core, then a two-way random tree classifier trained the software to distinguish tumor and non-tumor cells. Staining intensity for each tumor cell within a core was determined and assigned to an intensity group following manual calibration of thresholds (0 = none, 1 = weak, 2 = moderate, 3 = strong) with H-Score calculated as: [1×(% weakly stained) + 2×(% moderately stained) + 3×(% strongly stained)]. The mean tumor H-Score was calculated for each epithelioid patient with >2 scorable cores for BAP1/ASS1 (*n* = 164). These samples included 125 male and 33 female patients (6 no gender recorded) with a median age of 71 (range, 53–88); both gender and age distribution are in keeping with patient demographics.

### Bioinformatics and statistical analysis

Proteins modulated >1.5-fold in C5.1 *BAP1^w-/KO^* cells were functionally annotated using the WEB-based GEne SeT AnaLysis (GESA) Toolkit (http://www.webgestalt.org; RRID:SCR_006786; ref. 39) for GESA of Hallmark-50 pathways (40) and Over-representation analysis of Kyoto Encyclopedia of Genes and Genomes (KEGG) pathways. The Cancer Genome Atlas (TCGA) Pan-Cancer dataset for mesothelioma (10) was analyzed using cBioportal (https://www.cbioportal.org; RRID:SCR_014555; ref. 41) or exported for analysis. Morpheus (https://software.broadinstitute.org/morpheus; RRID:SCR_017386) was used to generate heatmaps and perform unsupervised hierarchical clustering.

Biochemical measurements represent several thousand cells and are represented as the mean value from at least three independent experiments, with error bars showing SD. No statistical method was used to predetermine sample size. All statistical tests were performed using GraphPad Prism version 9 (RRID:SCR_002798). Distribution of data was assessed by the D'Agostino & Pearson omnibus normality test and variance equivalency between samples by a variance homogeneity test. Data were analyzed by parametric or nonparametric tests as appropriate (see figure legends). In power calculations for TMA analyses, 200 patient samples were estimated to provide power of 75% to 80% to detect an OR of approximately 3.5, with 1:1–2:1 phenotypic ratio. Survival estimations were made using Kaplan–Meier method and plotted in IBM SPSS v26 (RRID:SCR_016479) or with Cox proportional hazards models in R v4 (RRID:SCR_017301; see Supplementary Materials and Methods). *P* values less than 0.05 were considered significant.

### Data availability

The data generated in this study are available within the article and its Supplementary Data. In this study, there are also analyses based upon datasets generated by TCGA Research Network (https://www.cancer.gov/tcga; RRID:SCR_003193).

## Results

To explore whether *BAP1* mutation leads to adaptations in MPM that may influence therapeutic strategies, we used genome editing to create isogenic cell lines with clinically relevant monoallelic and biallelic *BAP1* mutations on a mesothelial background. Transformed normal human pleural mesothelial MeT5A cells were selected as they have a near diploid genome (42). A clonal MeT5A-*BAP1^+/+^* parental line was established, which retains two copies of the *BAP1* locus (Supplementary Fig. S1) and expresses catalytically active BAP1 (Supplementary Fig. S2). Although SV40-transformed, MeT5A are not tumorigenic (42) and inactivation of P53 by the SV40 T-antigen is consistent with the genetic alteration of *CDKN2AB* or *TP53* commonly found in clinical MPM (9, 10). MeT5A-*BAP1^+/+^* therefore enabled sequential *BAP1* mutation, to generate a novel *in vitro* model of *BAP1* loss-of-function mutations in mesothelial cells. Genome editing with rAAV was used to first introduce a cancer-predisposition mutation on one *BAP1* allele, generating MeT5A-*BAP1^w-/+^* cells. This splice site A-to-G mutation, reported in the w-family germline, leads to exon 7 skipping, premature translational termination (Pro147fsX48), and nonsense mediated decay (15). A promoter trap was then introduced on the second *BAP1* allele to mimic biallelic inactivation in MPM, generating two independent MeT5A-*BAP1^w-/KO^* clones referred to as C3.1 and C5.1.

**Figure 1.**
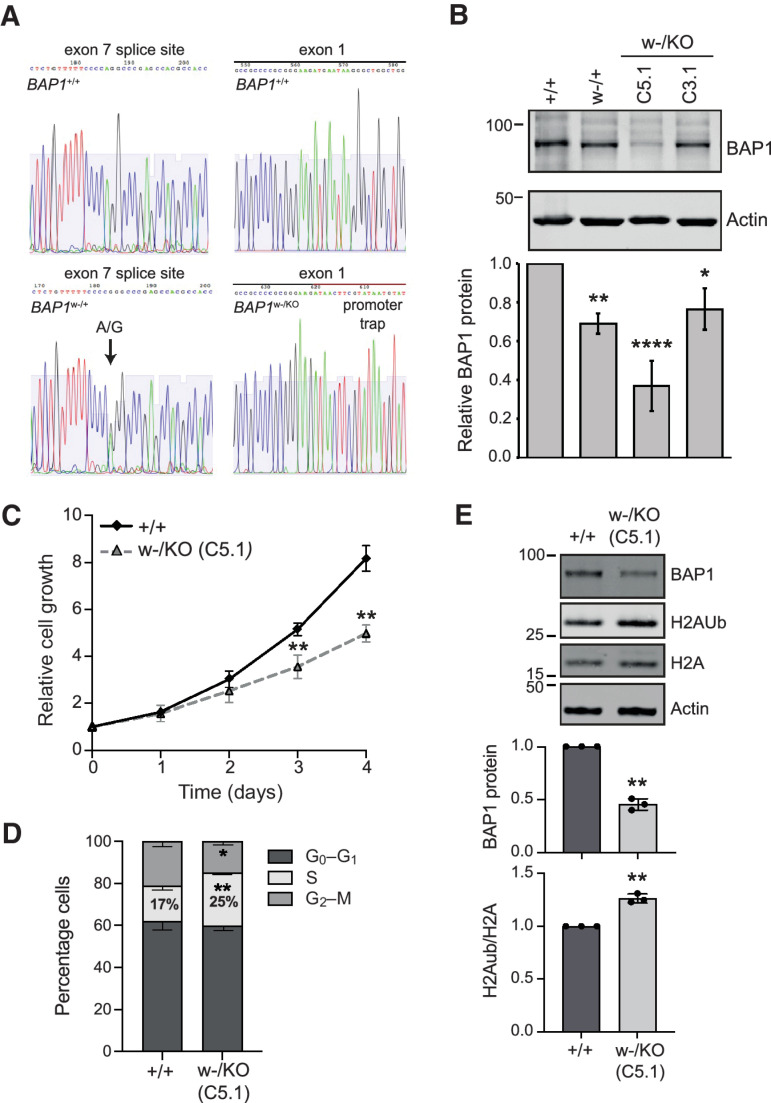
MeT5A *BAP1^w-/KO^* cells recapitulate phenotypic aspects of BAP1-deficient MPM cells. **A,** Sequence confirmation of edited alleles: introduction of the w-family splice site mutation to induce exon 7 skipping (left) and a promoter trap to knockout (KO) BAP1 expression (right) in the isogenic MeT5A cells. **B,** BAP1 protein levels are constitutively reduced in *BAP1^w-/+^* and *BAP1^w-/KO^* cells. Representative immunoblot and quantification relative to actin, mean of three independent experiments, error bars SD, one-way ANOVA with Dunnett *post hoc* test. *, *P* < 0.05; **, *P* < 0.01; ****, *P* < 0.0001. **C,** BAP1 deficiency slows proliferation of MeT5A cells. ATP-luciferase assay for *BAP1^w-/KO^* C5.1; mean of three independent experiments, error bars SD, *t* test. **, *P* < 0.01 compared with MeT5A-BAP1^+/+^. **D,** BAP1 deficiency causes MeT5A cells to accumulate in S-phase. Cell-cycle distribution determined by flow cytometry for *BAP1^+/+^* and *BAP1^w-/KO^* C5.1 (supporting data, Supplementary Fig. S5E). Mean of three independent experiments, error bars SD; unpaired *t* test; *, *P* = 0.0303; **, *P* = 0.0014. **E,** H2A ubiquitylation is increased in *BAP1*-mutated MeT5A cells. Representative immunoblot and quantification, mean of three independent experiments, error bars SD, one-sample *t* test. **, *P* < 0.01.

Targeted sequencing confirmed gene editing of each allele (Fig. 1A), while analysis of BAP1 transcripts confirmed altered splicing driven by the w-mutation and neomycin expression from within the promoter trap (Supplementary Fig. S3A and S3B). Surprisingly, the w-mutation increased overall *BAP1* transcription and only partially abrogated normal *BAP1* splicing (Supplementary Fig. S3C) so that mRNA encoding wild-type *BAP1* reduced by approximately 70% to 80% in *BAP1^w-/KO^* clones, with constitutive expression of catalytically active BAP1 protein reduced to approximately 60% in C3.1 and approximately 40% in C5.1, relative to parental MeT5A cells (Fig. 1B; Supplementary Fig. S3D). Of several thousand clones screened, only these two *BAP1^w-/KO^* clones correctly integrated the promoter trap. Consistent with identification of *BAP1* in CRISPR/Cas9 screens for fitness genes (17) and evidence that BAP1 loss promotes apoptosis on many cell backgrounds (18), our data suggest that complete deletion of *BAP1* may be incompatible with MeT5A cell survival. To investigate further, we performed whole-genome sequencing (WGS). This reconfirmed introduction of the correct *BAP1* mutations by genome editing and demonstrated little genomic divergence between cell lines (Supplementary Fig. S4; Supplementary Table S2). The number of differential SNPs relative to parental MeT5A cells was only 0.28% of that for MeT5A relative to the human reference genome. Between 16 and 19 differential SNPs were predicted to have potentially deleterious effects on an encoded protein, but only two were common between all cell lines (Supplementary Table S2). While we cannot rule out a contribution to survival of *BAP1*-mutated MeT5A cells, neither proteasome activator subunit 4 (PSME4) nor ATPase phospholipid transporting 11A (ATP11A) are obvious candidates.

**Figure 2.**
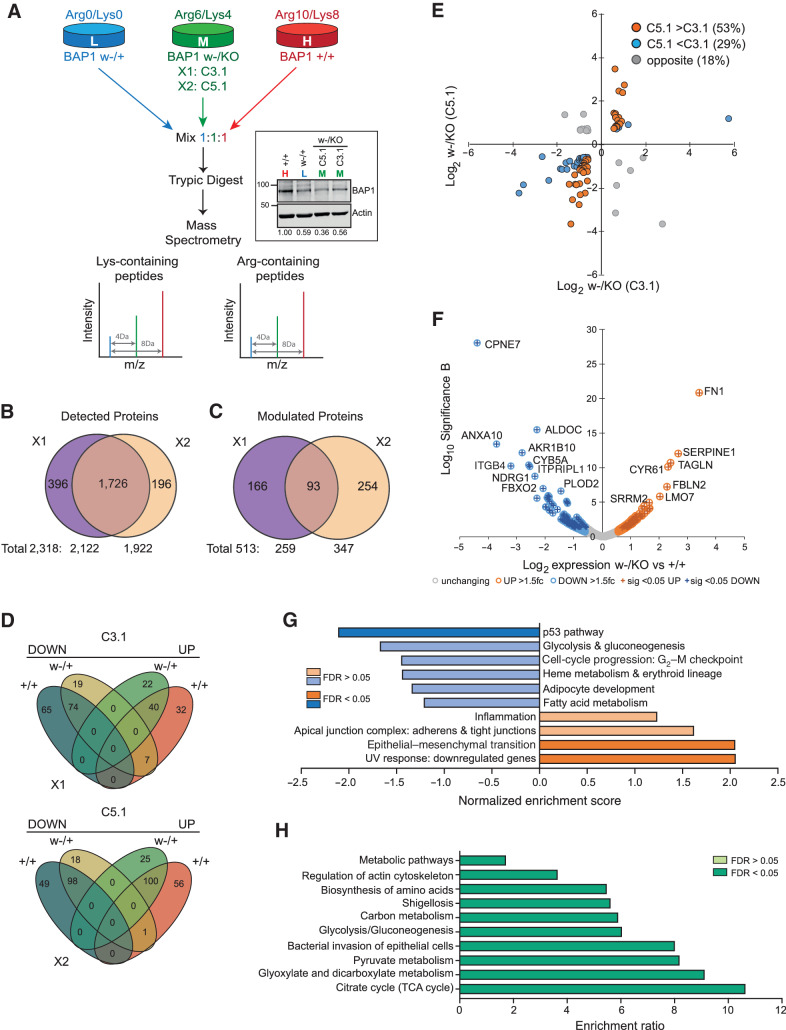
BAP1 deficiency reprograms the proteome of MeT5A cells. **A,** Experimental strategy for triplex SILAC-MS analysis of isogenic MeT5A cells, two alternative configurations (X1 and X2) used either the MeT5A-*BAP1^w-/KO^* C5.1 or C3.1 clones. Inset shows stable BAP1 expression after 6 passages in SILAC-labeling media; numbers indicate mean expression relative to *BAP1^+/+^* cells from three independent analyses. **B,** Summary of proteins identified by MS across two experiments, based on gene name identifiers; supporting data, Supplementary Table S3. **C,** Summary of proteins modulated >1.5-fold in *BAP1^w-/KO^* relative to either *BAP1^+/+^* or *BAP1^w-/+^* cells. **D,** Overview of directional expression changes in each experimental configuration for the 513 proteins modulated by >1.5-fold. **E,** Relative expression of the 93 proteins modulated >1.5-fold in both *BAP1^w-/KO^* clones. **F,** Volcano plot showing proteins modulated >1.5-fold (colored circles) with *P* < 0.05 (crossed circles) in C5.1 *BAP1^w-/KO^* cells. **G** and **H,** Heatmaps summarizing functional annotation of enriched pathways for proteins modulated by >1.5-fold in C5.1 *BAP1^w-/KO^* cells. Gene set enrichment analysis showing weighted set cover for Hallmark-50 pathways: blue, C5.1 downregulated proteins; orange, C5.1 upregulated proteins (**G**); supporting data in Supplementary Fig. S7A and S7B. Overrepresentation analysis for KEGG pathways among all C5.1 modulated proteins (**H**).

Importantly, *BAP1^w-/KO^* clones phenocopied the effect of BAP1 depletion on growth characteristics of MPM cells (14), with reduced proliferation and accumulation in S-phase (Fig. 1C and D; Supplementary Fig. S5). We also observed moderately increased ubiquitylation of histone H2A K119, a major and abundant target of BAP1 (18), in the *BAP1^w-/KO^* C5.1 cells commensurate with their residual BAP1 (Fig. 1E). To further explore the effects of BAP1 deficiency, we used SILAC-MS in two triplex-configured experiments to compare the proteome of the MeT5A isogenic cell panel (Fig. 2A). Overall, we identified more than 2,300 individual proteins (Supplementary Table S3), 75% of which were detected in both experiments (Fig. 2B). Compared with either parental or haploinsufficient cells, 22% of detected proteins increased or decreased by more than 1.5-fold in the *BAP1^w-/KO^* clones (Fig. 2C), with sequential gene editing leading to stepwise divergence in the proteome (Fig. 2D). Consistent with residual BAP1 levels in the *BAP1^w-/KO^* clones (Fig. 2A), more proteins were modulated in C5.1 than C3.1 (Fig. 2D) and most of the proteins modulated in both *BAP1^w-/KO^* clones showed greater differential expression in C5.1 (Fig. 2E). We therefore focused on investigating adaptation of *BAP1^w-/KO^* C5.1 cells compared with *BAP1^+/+^* cells (Fig. 2F).

Functional annotation of proteins modulated in *BAP1^w-/KO^* C5.1 cells revealed marked enrichment of gene ontology (GO) terms and pathways involved in cancer cell biology, metabolism, and movement of cells (Fig. 2G and H; Supplementary Fig. S6). None of the 18 differential deleterious SNPs identified by WGS in the *BAP1^w-/KO^* C5.1 clone mapped to relevant pathways or biological processes (Supplementary Table S2). GESA identified hallmark pathways associated with cancer including cell-cycle progression, the P53 pathway, metabolism, and epithelial–mesenchyme transition (EMT; Fig. 2G; Supplementary Fig. S7A and S7B), highlighting the disease relevance of our *BAP1^w-/KO^* mesothelial cell model. Highly modulated EMT markers included fibulin 2 (FBLN2), a paralog of the MPM biomarker FBLN3 (43), and transglutaminase 2 (TGM2), a cancer stem cell survival protein required for MPM tumor formation (44). Indeed, fibronectin (FN1) and plasminogen activator inhibitor 1 (SERPINE1, PAI), the most significantly upregulated proteins in *C5.1 BAP1^w-/KO^* cells (Fig. 2F), are associated with EMT. However, while FN1 and PAI1 validated by immunoblotting (Supplementary Fig. S7C–S7E), they did not correlate with BAP1 status in an MPM cell panel (Supplementary Fig. S7F–S7H). We therefore focused our attention on the metabolic reprogramming highlighted by both pathway and GO term analyses. Glycolysis/gluconeogenesis and tricarboxylic acid (TCA) cycle KEGG pathways were overrepresented (Fig. 2H), with proteins downregulated in C5.1 enriched for glycolysis/gluconeogenesis (Fig. 2G; Supplementary Fig. S7A) and those upregulated in C5.1 enriched for TCA metabolic processes (Supplementary Fig. S6).

**Figure 3.**
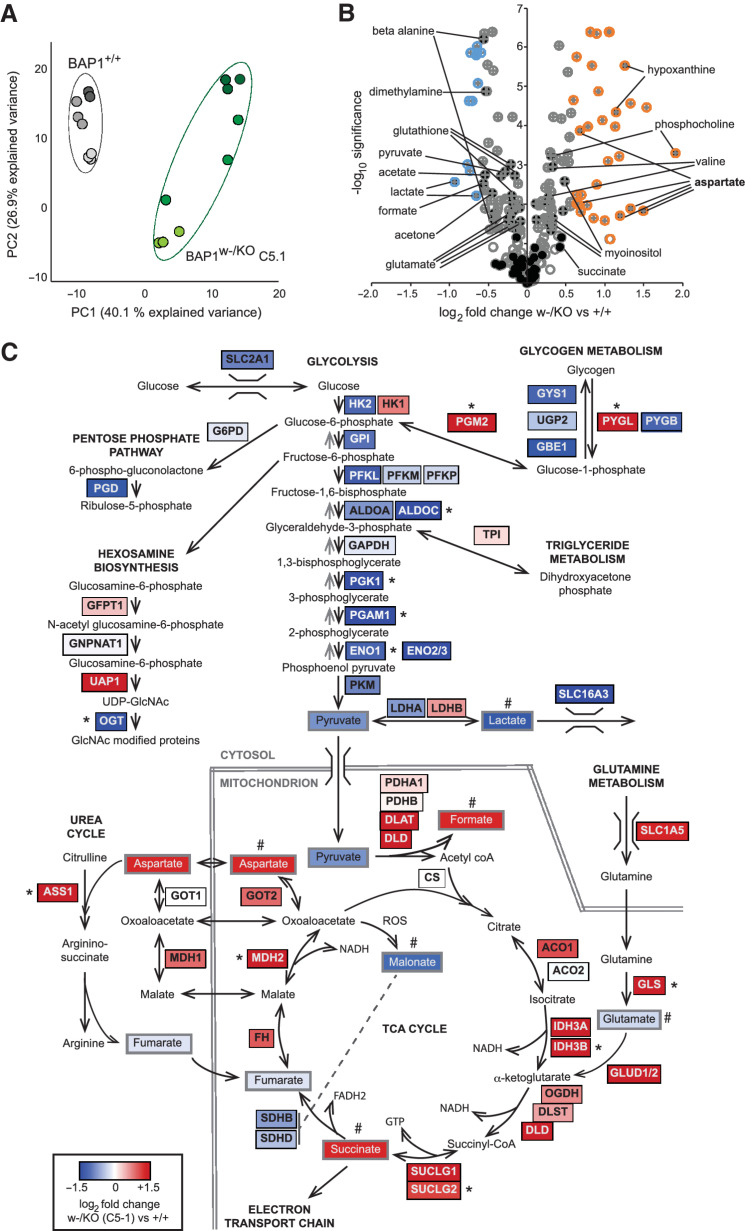
BAP1-deficient MeT5A cells have altered levels of metabolites and metabolic enzymes. **A** and **B,** NMR analysis reveals altered metabolites in C5.1 *BAP1^w-/KO^* relative to *BAP1^+/+^* MeT5A. PCA plot for samples from three independent experiments analyzed in triplicate (**A**). Volcano plot of metabolites modulated >1.5-fold (colored circles) with *P* < 0.05 (crossed circles), named metabolites (filled black circles; **B**). **C,** Overview of metabolic adaptation for key pathways in BAP1-deficient MeT5A cells combining proteomic and metabolomic data, color scale represents log_2_ fold change in C5.1 MeT5A-*BAP1^w-/KO^* versus *BAP1^+/+^* cells. *Enzymes investigated further. ^#^Significantly modulated metabolites (supporting data, Supplementary Table S4; Supplementary Fig. S8).

**Figure 4.**
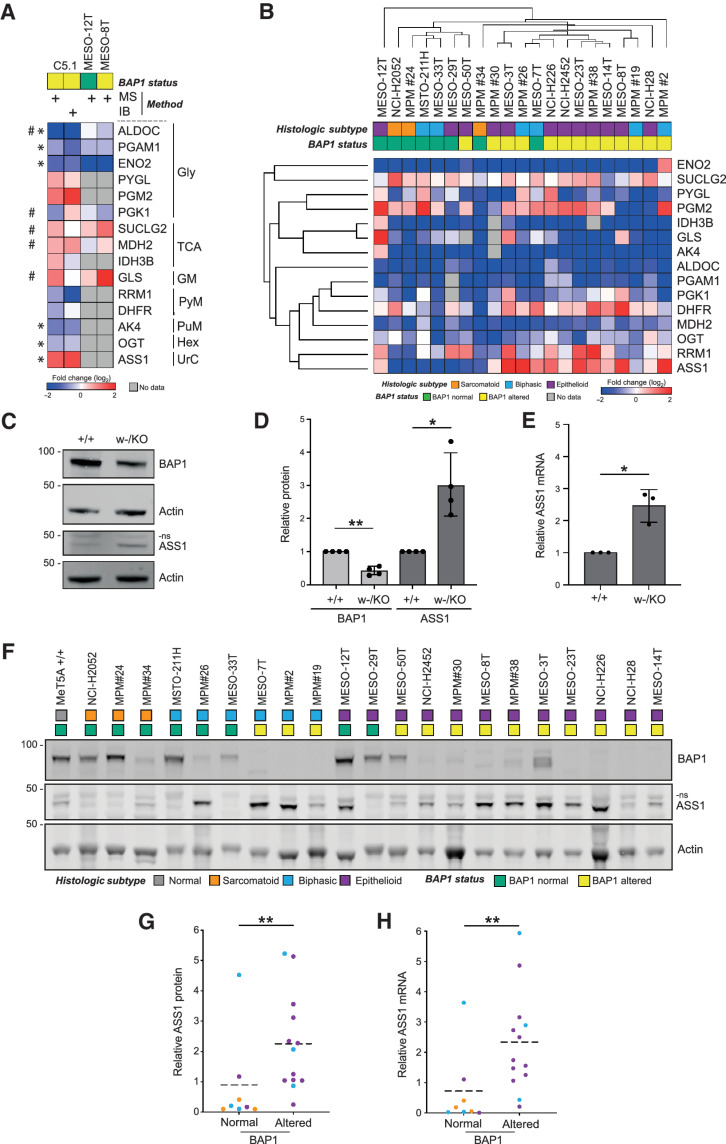
ASS1 expression is a metabolic BAP1 dependency in isogenic MeT5A and MPM cell lines. **A,** Heatmap comparing expression in *BAP1^w-/KO^* C5.1 cells determined by SILAC-MS or immunoblotting (IB, three independent experiments, supporting data in Supplementary Fig. S9), with SILAC-MS data (*n* = 1; supporting data, Supplementary Table S3) for two epithelioid MPM cell lines: MESO-8T (BAP1-altered), MESO-12T (BAP1-normal). *Significant modulation confirmed in MeT5A by immunoblotting, ^#^data for MESO-8T versus MESO-12T consistent with BAP1 dependency in MeT5A. Gly, glycolysis; TCA, tricarboxylic acid cycle; GM, glycogen metabolism; PyM, pyrimidine metabolism; PuM, purine metabolism; Hex, hexosamine biosynthesis; UrC, urea cycle. **B,** Unsupervised hierarchical clustering for expression in 21 MPM cell lines determined by immunoblotting. Heatmap shows protein levels for selected metabolic enzymes in cell lines relative to MeT5A *BAP1^+/+^*. Key indicates histologic subtype and BAP1 status, supporting data in Supplementary Figs. S10 and S11. ASS1 protein levels increase in MeT5A *BAP1*^w-/KO^ cells. Representative immunoblot with anti-ASS1 antibody (Millipore; **C**) and quantification (**D**). Mean of four independent experiments, error bars SD; Welch *t* test; *, *P* = 0.026; **, *P* = 0.003; ns, non-specific band. **E,***ASS1* mRNA expression increases in MeT5A *BAP1*^w-/KO^ cells. *ASS1* qRT-PCR normalized to *ACTB* and *GAPDH*; mean of three independent experiments, error bars SD; Welch *t* test; *, *P* = 0.021. **F–H,** ASS1 protein and mRNA expression are significantly higher in BAP1-altered than BAP1-normal MPM cell lines. Representative immunoblot for the cell panel (**F**); histologic subtype and BAP1 status indicated; ns, non-specific band. Mean ASS1 protein level from three independent experiments (**G**); normalized to actin and relative to MeT5A *BAP1*^+/+^, population mean (dashed line), Mann–Whitney **, *P* = 0.006. Mean *ASS1* mRNA determined by qRT-PCR (**H**); normalized to *ACTB* and *GAPDH* relative to MeT5A *BAP1*^+/+^ from three independent experiments, population mean (dashed line), Mann–Whitney **, *P* = 0.006. For **G** and **H,** histologic subtype is indicated in **F**; supporting data, Supplementary Fig. S12.

To investigate further, we interrogated *BAP1^w-/KO^* and *BAP1^+/+^* MeT5A cells using NMR metabolomics (Supplementary Table S4). PCA showed clear separation of the cell lines according to PC1, which accounted for 40% of the variance among samples (Fig. 3A), and 82 metabolite peaks in the NMR spectra were formally identified (Fig. 3B). To integrate protein and metabolite data, we mapped both datasets onto key metabolic pathways (Fig. 3C). In *BAP1^w-/KO^* cells, differential expression of around 60 proteins was associated with the most enriched metabolic processes. Particularly striking was reduced expression of multiple glycolysis/gluconeogenesis enzymes, accompanied by downregulation of glucose and lactate transporters. Notably, levels of lactate and pyruvate were also low, potentially consistent with reduced glycolysis (Fig. 3B and C; Supplementary Fig. S8). In contrast, there was increased expression of enzymes promoting glycogen or glutamine metabolism, the TCA cycle and anaplerotic pathways, including the urea cycle. Correspondingly, levels of identified TCA intermediates were also higher in *BAP1^w-/KO^* MeT5A and aspartate, which feeds into the urea cycle, was prominently upregulated (Fig. 3B and C; Supplementary Fig. S8). For selected metabolic enzymes identified by MS, their expression was cross-validated by immunoblotting in independent unlabeled MeT5A lysates (Fig. 4A, compare columns 1 and 2). In general, where protein expression decreased in *BAP1^w-/KO^* cells by MS, this was confirmed by immunoblotting, including for the glycolysis enzymes ALDOC, PGAM1, and ENO2, as well as AK4 and O-linked N-acetylglucosamine transferase (OGT; Fig. 4A; Supplementary Fig. S9). PGM2 trended toward higher expression in *BAP1^w-/KO^* cells (Fig. 4A; Supplementary Fig. S9) while ASS1 levels were most significantly increased (Fig. 4A). Thus, metabolic reprogramming in *BAP1^w-/KO^* MeT5A may signal reliance on alternative energy sources and anaplerotic pathways.

To determine whether metabolic adaptions to *BAP1* mutation in the isogenic MeT5A model were also evident in BAP1-altered MPM, we utilized a panel of 21 patient-derived cell lines, representing the histologic and genetic heterogeneity of the disease. These were characterized for BAP1 expression, catalytic activity, and cellular localization, with confirmatory targeted sequencing where indicated, to stratify the panel according to BAP1 status (Supplementary Fig. S10). Two epithelioid cell lines were chosen that differed in BAP1 status but were otherwise closely matched in terms of the originating laboratory, growth media, presence of MPM markers and lack of NF2/CDKN2A expression. The MESO-8T (BAP1-altered) and MESO-12T (BAP1-normal) cells were initially compared with parental *BAP1^+/+^* MeT5A cells by triplex SILAC-MS (Supplementary Table S3; Fig. 4A columns 3 and 4). Seven metabolic enzymes of interest were detected in both MPM cell lines, with the majority showing the highest differential expression in MESO-8T, consistent with BAP1 dependency predicted from the MeT5A model (Fig. 4A).

As it is difficult to generalize from two cell lines, fuller coverage of metabolic enzymes of interest across the MPM cell panel was obtained by immunoblotting (Fig. 4B; Supplementary Fig. S11A). Levels of several enzymes (e.g., ALDOC, PGAM1, ENO2) were consistently reduced in all MPM cell lines, irrespective of BAP1 status, compared with the normal mesothelial *BAP1^+/+^* MeT5A (Fig. 4B). Intriguingly *BAP1^w-/KO^* MeT5A cells largely recapitulate these cancer-associated expression changes (Fig. 4A) suggesting *BAP1* mutation may be sufficient, although not required, for adaptation. Metabolic enzymes including OGT, DFHR, and RRM1 exhibited variable expression amongst the MPM cell lines (Fig. 4B). DHFR and RRM1 are linked with sensitivity to therapeutics used in MPM, pemetrexed, and gemcitabine, respectively; however, they did not stratify by BAP1 status (Supplementary Fig. S11B), implying more complex regulation of their expression in cancer. We did however observe a striking correlation between BAP1 status and levels of ASS1 (Fig. 4B), an essential enzyme in arginine synthesis that may predict sensitivity to certain therapeutics targeting metabolism. We also examined the relationship between expression of these metabolic transcripts and *BAP1* genetic alteration, transcript expression, or protein levels in TCGA MESO dataset (ref. 10; Supplementary Fig. S11C). Although limited association was evident for transcripts including *OGT*, only *ASS1* significantly correlated with all three molecular measures of BAP1 status, reinforcing data from the MPM cell lines.

We investigated the BAP1 dependence of ASS1 expression in more depth using two validated antibodies (Supplementary Fig. S12A and S12B). Significantly higher levels of ASS1 protein were confirmed in *BAP1^w-/KO^* compared with the parental *BAP1^+/+^* MeT5A cell line (Fig. 4C and D; Supplementary Fig. S12C and S12D). Analysis of *ASS1* mRNA in parallel samples demonstrated that this was driven by a transcriptional response to *BAP1* mutation (Fig. 4E). Similarly, both protein and mRNA expression of ASS1 were significantly higher in MPM cell lines with altered BAP1 (Fig. 4F–H; Supplementary Fig. S12E–S12G). *ASS1* mRNA and protein were highly correlated across the cell lines (Supplementary Fig. S12H) suggesting that transcript expression is a useful surrogate for protein levels. Consistent with a transcriptional response to BAP1 inactivation, *ASS1* mRNA expression was also inversely correlated with that of *BAP1* in the MPM cell lines (Supplementary Fig. S12I).

**Figure 5.**
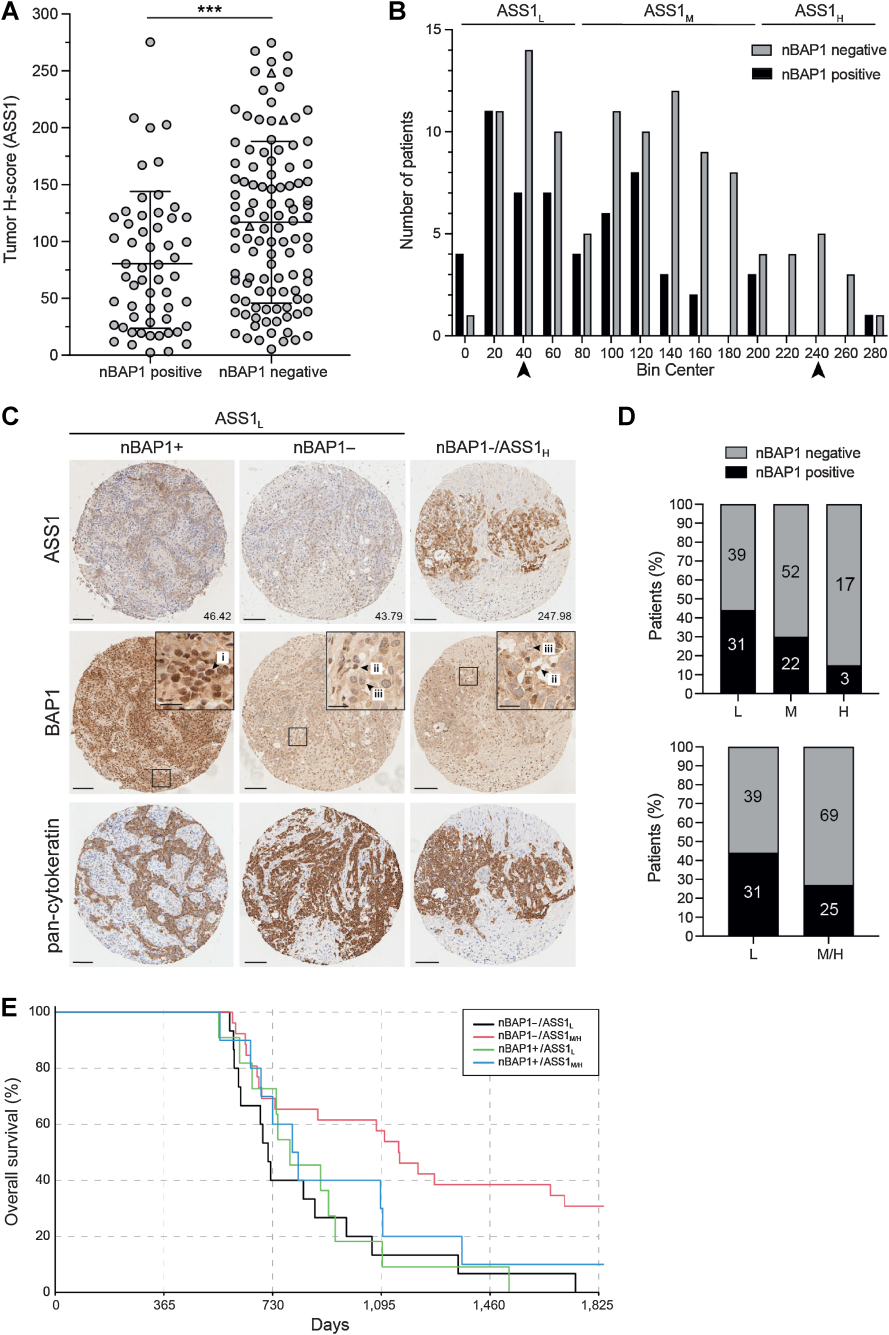
ASS1 protein levels are elevated in epithelioid MPM samples with loss of nBAP1. nBAP1 staining was scored as positive (*n* = 56) or negative (*n* = 108) by three independent observers, and mean ASS1 tumor H-scores digitally determined for 164 epithelioid samples (2 to 4 cores per patient). **A,** ASS1 expression is higher in epithelioid MPM without nBAP1; bars indicate mean and 1 SD; Mann–Whitney test, ***, *P* = 0.001. **B,** Frequency distribution of ASS1 tumor H-scores defines three groups: ASS1_L_ (low ASS1, tumor H-score <80); ASS1_M_ (moderate ASS1, tumor H-score ≥80–200); ASS1_H_ (high ASS1, tumor H-score ≥200). **C,** Images of MPM cores representing the mid-point of the ASS1_L_ and ASS1_H_ groups (indicated by arrow heads in **B**). The tumor H-score for ASS1 is shown; magnified insets indicate (i) nBAP1-positive tumor cells, (ii) nBAP1-positive stromal cells, (iii) nBAP1-negative tumor cells. Scale bars, 100 or 20 μm in insets. **D,** Loss of nBAP1 is more frequent in groups with higher ASS1 expression. Comparison of the three ASS1 groups *χ*^2^ test, ***, *P <* 0.0001 (top); and ASS1 _L_ group compared to the combined ASS1_M/H_ groups, Fisher exact test, *, *P* = 0.018 (below). The numbers of nBAP1-positive or nBAP1-negative patients in each group are indicated. **E,** Improved survival of patients with nBAP1-negative ASS1_M/H_ tumors. Kaplan–Meier curve is shown at 18-month landmark. Cox model: nBAP1-negative ASS1_M/H_ (*n* = 26) versus ASS1_L_ (*n* = 15) *P* = 0.003; nBAP1-positive ASS1_M/H_ (*n* = 10) versus ASS1_L_ (*n* = 11) *P* = 0.383; supporting data in Supplementary Table S5 and Supplementary Fig. S13.

To further explore this relationship, ASS1 protein levels were assessed in MPM tissue samples, which were also stained for nBAP1, as a surrogate for *BAP1* mutation and loss of function, as well as pan-cytokeratin as a tumor cell marker (Fig. 5). In line with previous studies (11–13), loss of nBAP1 was most common among patients with epithelioid MPM in our cohort. Given the confounding effects of histologic subtypes for digital pathology and the relative frequency of *BAP1* mutations, we focused on the 164 epithelioid cases, 66% of which lacked nBAP1 (Supplementary Table S5). Digital quantification of total cellular ASS1 protein revealed significantly higher ASS1 in tumors without nBAP1 (*n* = 108) compared with those with nBAP1 (*n* = 56, Mann–Whitney *P* = 0.001; Fig. 5A). The tumor H-score distribution defined three groups as ASS1 low (ASS1_L_), moderate (ASS1_M_), or high (ASS1_H_). The proportion of nBAP1-negative cases significantly increased across these groups, so that ASS1_H_ cases were almost exclusively nBAP1-negative (Fig. 5B–D).

While there were no statistically significant differences in overall survival (OS) between nBAP1-positive and -negative groups, or based on ASS1 H-score, we observed a late separation in the curves (Supplementary Fig. S13A–S13D). Therefore, further landmark analyses were performed at 12, 18, and 24 months, where differences in survival with ASS1 H-score were evident (Supplementary Table S5; Supplementary Fig. S13E–S13G) consistent with previous reports (6). Notably however, the best outcomes were seen for BAP1-negative ASS1_M/H_ cases (Fig. 5E; Supplementary Table S5). This was a small subgroup of patients with a very late separation in the curves and does not suggest a clinically significant difference in outcome. However, the cosegregation of the biomarkers suggests a potentially distinct biology for this subgroup of MPM.

**Figure 6.**
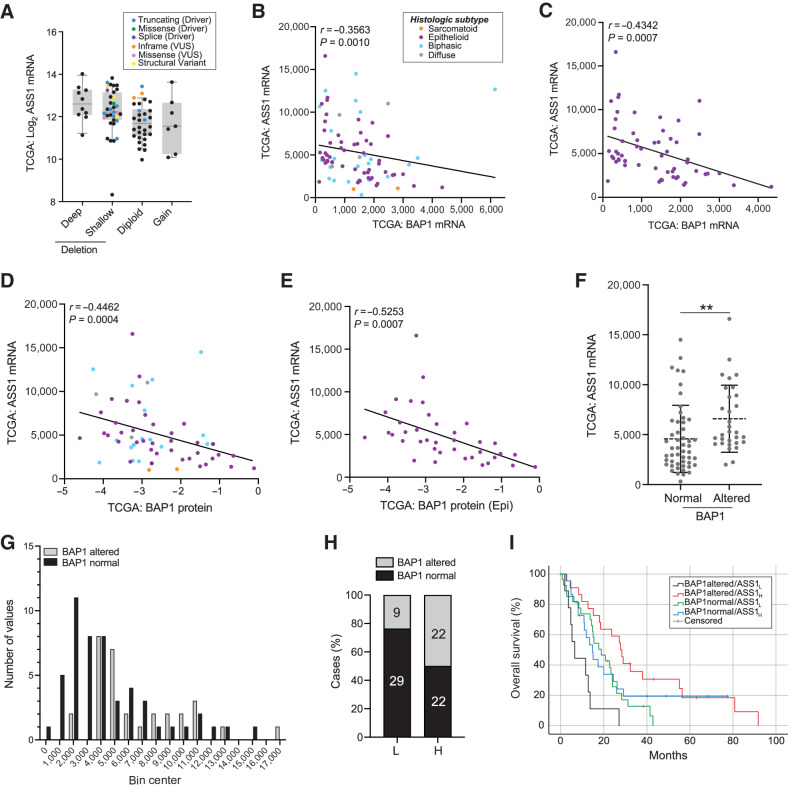
Association between BAP1 status, *ASS1* transcript expression and survival in TCGA MESO cohort. **A,***ASS1* transcript levels are higher in cases with deletion and/or mutation of *BAP1*. Putative *BAP1* copy-number variations from GISTIC; *ASS1* mRNA expression log_2_ batch normalized RSEM, colored by BAP1 mutation. **B–E,** Inverse correlation of *ASS1* mRNA (batch normalized RSEM) with BAP1 colored by histologic type as indicated in **B**. *BAP1* mRNA (for the cohort, *n* = 82 (**B**) or only the epithelioid cases, *n* = 58 (**C**), and BAP1 protein (RPPA) for the cohort, *n* = 59 (**D**) or only the epithelioid cases, *n* = 38 (**E**); Spearman correlations shown. **F,***ASS1* mRNA is higher in *BAP1*-altered cases (deletion and/or mutation); *n* = 82, bars indicate mean and 1 SD; Mann–Whitney test, **, *P* = 0.0016. **G,** Frequency distribution of *ASS1* mRNA stratified by *BAP1* status. **H,***BAP1* alteration is more frequent in tumors with higher *ASS1* expression. *ASS1* transcript stratified by z-scores: below zero (*ASS1*-low) or above zero (*ASS1*-high); Fisher exact test, ****, *P* = 0.0002; the numbers of *BAP1*-normal or *BAP1*-altered patients in each group are indicated. **I,** Improved survival of patients with *BAP1*-altered *ASS1*-high tumors. Kaplan–Meier analysis for 69 uncensored patients according to groups in **H**. Median survival (lower and upper bound): *BAP1*-altered *ASS1*-high 21.6–33.9 months (*n* = 19), *BAP1*-altered *ASS1*-low 2.7–10.2 months (*n* = 9), *BAP1*-normal *ASS1*-high 10.1–19.3 months (*n* = 17), *BAP1*-normal *ASS1*-low 11.8–25.8 months (*n* = 24); Log-rank (Mantel–Cox) *P* = 0.001. The results shown in this figure are based upon data generated by TCGA Research Network: https://www.cancer.gov/tcga.

To explore whether the relationship between ASS1 and BAP1 was a general feature of cancer, we examined TCGA pan-cancer datasets. Although most significant in MPM, inverse correlation was evident in the other cancers with common *BAP1* inactivation (cholangiocarcinoma, uveal melanoma, and RCCC) but relatively uncommon among other cancers (Supplementary Fig. S14), suggesting cell context specificity. As *ASS1* transcript inversely correlated with *BAP1* genetic alteration, transcript expression, and protein levels in TCGA MESO dataset, particularly among the epithelioid subtype (Fig. 6A–E), we used this independent cohort to confirm findings from our IHC study. On stratification by BAP1 status, *ASS1* transcript was significantly higher in BAP1-altered tumors (Mann–Whitney, *P* = 0.0016; Fig. 6F), while the distribution of *ASS1* expression was similarly shifted for BAP1-altered cases in both cohorts (Figs. 5B and 6G). Further stratification of TCGA MESO cohort by *ASS1* Z-scores showed marked enrichment of BAP1-normal cases within the ASS1-low group (Fisher exact test *P* = 0.0002; Fig. 6H). Notably, the BAP1-altered ASS1-low cases had the worst outcomes in TCGA cohort, with longer survival for BAP1-altered ASS1-high cases (Fig. 6I) similar to our IHC study (Fig. 5E).

BAP1 IHC is robust, binary, and increasingly adopted into routine clinical pathology, facilitating its use to inform patient care (13). This presents an opportunity to use BAP1 IHC to help stratify patients for therapeutic approaches that target ASS1 or downstream metabolic pathways, or that cause arginine deprivation. In other cancers, high ASS1 is associated with poor prognosis and increased purine synthesis, potentially sensitizing cells to mizoribine (45). However, in MPM, we find high ASS1 predominantly associated with nBAP1 loss and better prognosis (Fig. 5). Indeed, *BAP1^w-/KO^* MeT5A cells with elevated ASS1 were more resistant to mizoribine (Supplementary Fig. S15A). In MPM cell lines, the LC_50_ ranged from 9.7 μmol/L to >1.2 mmol/L (Supplementary Fig. S15B); however, mizoribine sensitivity appeared unrelated to ASS1 expression or BAP1 status, correlating most closely with proliferative capacity (Supplementary Fig. S15C) and suggesting MPM are biologically distinct from other ASS1-high cancers (45).

ASS1 function is blocked by αMDLA which can reduce growth of lung or colorectal cancer cells with high ASS1 (45, 46). Although elevated in *BAP1^w-/KO^* MeT5A, the *BAP1^+/+^* counterpart also expresses ASS1, and treatment with αMDLA similarly reduced growth of both cell lines (Supplementary Fig. S16A and S16B). This suggests MeT5A mesothelial cells may be able to utilize extracellular arginine when ASS1 is inhibited. To assess utility of αMDLA in MPM cancer cells, we selected the paired MESO-8T (BAP1-altered, ASS1-moderate) and MESO-12T (BAP1-normal, ASS1-low) cells, and another pair of low passage epithelioid cells that have similar tumor suppressor status (lacking CDKN2A but not NF2, Supplementary Fig. S10) and mizoribine sensitivity (Supplementary Fig. S15): MESO-29T (BAP1-normal, ASS1-undetectable) and MESO-23T (BAP1-altered, ASS1-moderate). The BAP1-altered MPM cell lines appeared moderately more sensitive to αMDLA when grown in full media. A significant reduction in cell confluency was visible after treatment with 10 mmol/L αMDLA, a dose commonly used in the literature, although an ATP-dependent viability assay appeared less reliable in this context (Supplementary Fig. S16C–S16F).

**Figure 7.**
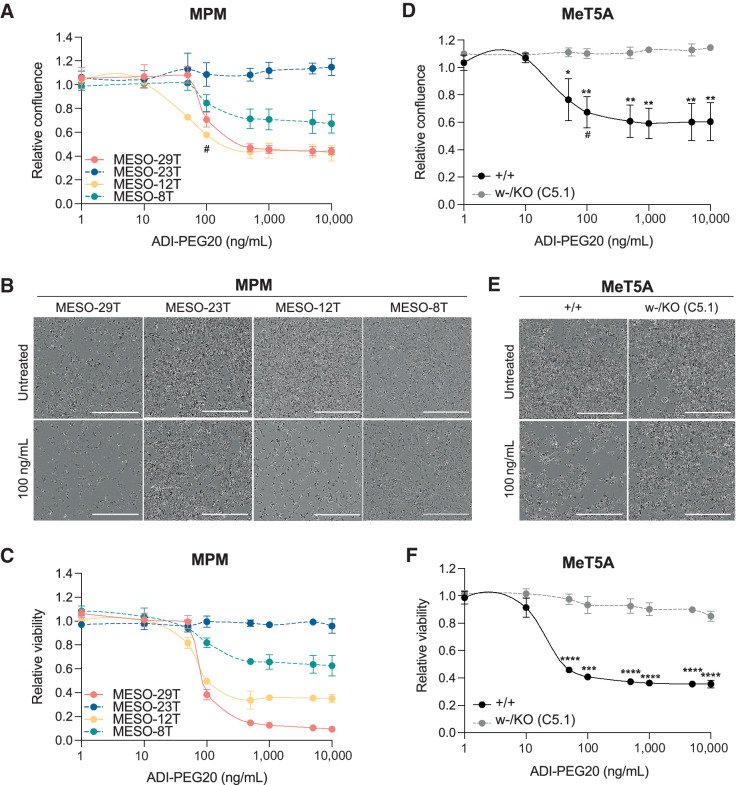
The influence of BAP1 status on response to arginine deprivation with ADI-PEG20. Paired MPM cell lines MESO-29T (BAP1-normal/ASS1-low) and MESO-23T (BAP1-altered/ASS1-high), MESO-12T (BAP1-normal/ASS1-low) and MESO-8T (BAP1-altered/ASS1-high; **A–C**) or MeT5A *BAP1*^+/+^ and *BAP1*^w-/KO^ cells (**D–F**) were treated for 96 hours with ADI-PEG20; mean of three independent experiments, error bars SD; *t* test; *, *P* < 0.05; **, *P* < 0.01; ***, *P <* 0.001; ****, *P* < 0.0001. Dose response for relative confluence assessed by live imaging at 96 hours (**A** and **D**), # indicates 100 ng/mL ADI-PEG20, the approximate LC_50_ for sensitive cells, for which representative images at 96 hours are shown (**B** and **E**). ATP-based luciferase assay conducted following imaging at 96 hours (**C** and **F**). Summary data, Supplementary Fig. S16F.

In an inverse approach, arginine deprivation by ADI-PEG20 is undergoing clinical trials in ASS1-deficient cancers that depend upon uptake of exogenous arginine, including nonepithelioid MPM (6). As expected, the BAP1 normal cell lines MESO-29T and MESO-12T were sensitive, while BAP1-altered cell lines MESO-23T and MESO-8T were more resistant (Fig. 7A–C). Overall, the MPM cell lines showed inverse sensitivity to αMDLA and ADI-PEG20, that related to their ASS1 expression and BAP1 status (Supplementary Fig. S16F). To investigate whether *BAP1* mutation influenced ADI-PEG20 sensitivity, we compared dose responses in the isogenic MeT5A cells (Fig. 7D–F). *BAP1^+/+^* MeT5A were sensitive to ADI-PEG20 with a similar LC_50_ to MESO-29T, while strikingly *BAP1^w-/KO^* MeT5A were resistant to ADI-PEG20, phenocopying MESO-23T. Thus, *BAP1* mutation is sufficient to induce resistance, suggesting that BAP1 status may be driving the survival benefit previously reported in patients with epithelioid MPM treated with ADI-PEG20 (6).

## Discussion

BAP1 loss of function is an early event in MPM tumorigenesis and defines a molecular subtype of MPM with likely distinct drug sensitivities. A number of previous studies, mainly utilizing MPM cell lines with differing BAP1 status, have suggested specific sensitivities, for example targeting TNF-related apoptosis inducing ligand (TRAIL; ref. 28), FGFR (47), histone deacetylases (HDAC; ref. 32), EZH2 (48) or more controversially PARP (29, 49). However, to date relevant agents have typically shown either poor efficacy or limited association with BAP1 status in clinical trials. For example, a recent trial of the PARP inhibitor rucaparib reported benefits for some patients with MPM, but this was not predicted by BAP1 status (31). While the reasons remain opaque, this is likely in part due to heterogeneity of cell lines with the presence of confounding mutations and adaptations. We therefore sought to develop an isogenic model on a mesothelial background to remove as many confounding factors as possible. We knocked in a mutation that predisposes to familial mesothelioma (15) and asbestos-induced tumors in mice (24), combined with knockout of the other *BAP1* allele. MeT5A-*BAP1^w-/KO^* have stably reduced BAP1 expression and, given that *BAP1* mutation is an early truncal change (8), represent a more relevant model to study tumor biology and drug sensitivity than modulation of BAP1 in established cancer cell lines that already harbor a spectrum of tumorigenic mutations.

We profiled MeT5A-*BAP1^w-/KO^* cells by SILAC-MS to investigate alternative BAP1 dependencies that may confer therapeutic sensitivity. No protein expression changes were evident amongst epigenetic regulators or DNA repair processes, instead cytoskeletal organization and metabolic processes were significantly enriched. Similar pathways were identified by proteomics following reintroduction of BAP1 into *BAP1*-null NCI-H226 lung cancer cells (27); however, the specific proteins and cellular responses differ, reinforcing the context-specific nature of BAP1 function.

Among the enriched metabolic pathways, downregulation of glycolysis enzymes and upregulation of TCA cycle enzymes were evident in MeT5A-*BAP1^w-/KO^*. However, for most of these proteins, their levels were not closely linked to BAP1 status in MPM cell lines, suggesting potential convergent evolution during tumorigenesis. Interestingly, glycolytic enzymes were mostly reduced compared with wild-type MeT5A in both MeT5A-*BAP1^w-/KO^* and the MPM cell lines. While functional analysis would be required to draw firm conclusions, this appears to be at odds with the Warburg effect, where cancer cells rely more heavily on glycolysis for energy production. Germline *BAP1* mutation was previously shown to induce a Warburg effect in fibroblasts from affected *BAP1^+/−^* individuals (50), but in contrast an *in vivo* proteomic study of Neucode-labeled *Bap1* knockout mice revealed metabolic changes that included decreased glycolysis/gluconeogenesis (51). Interestingly, OGT, which posttranslationally modifies proteins by addition of N-Acetylglucosamine (GlcNAc), was downregulated in MeT5A-*BAP1^w-/KO^* cells. OGT was previously identified as an interactor and substrate of BAP1 (52) that by modifying PGC-1α with GlcNAc, facilitates its stabilization by BAP1 to promote gluconeogenesis (53). Thus, BAP1 can interplay with glycolysis/gluconeogenesis with context-specific consequences.

The most notable novel BAP1 dependency identified in our study was ASS1, an enzyme essential for cellular synthesis of arginine. MeT5A-*BAP1^w-/KO^* cells had elevated ASS1 mRNA and protein, which was recapitulated in BAP1-altered patient-derived MPM cell lines and tissues samples. In all cases, this appears to be a transcriptional response to BAP1 loss, implying that BAP1 normally represses *ASS1* transcription, either directly or indirectly. While we have not formally demonstrated higher ASS1 enzymatic activity, it is generally accepted that increased transcription of *ASS1* is indirect evidence for the cellular requirement to generate argininosuccinate endogenously. BAP1 operates as a transcriptional activator through its role in deubiquitylating H2A (18), so likely regulates *ASS1* through an alternative mechanism. Although defining this is beyond the scope of the current study, we found no evidence for BAP1 recruitment to the *ASS1* gene promoter in publicly available chromatin immunoprecipitation sequencing data, and one possibility is that BAP1 interplays with the methylation of CpG islands that restrict *ASS1* expression (6).

ASS1 is often silenced in cancers, including MPM, conferring reliance upon extracellular arginine, and hence susceptibility to arginine deprivation using ADI-PEG20. However, elevated *ASS1* mRNA and protein have also been reported in MPM three-dimensional spheroid models and patient samples (54). We confirm that ASS1 can be highly expressed in MPM and show for the first time that this is often associated with BAP1 loss. This elevated ASS1 expression was associated with resistance to ADI-PEG20, an indirect measure of ASS1 enzymatic activity. In some cancers, high ASS1 is associated with poor prognosis (45); however, like BAP1 loss (22), elevated ASS1 is associated with better OS in patients with MPM (6). This was recapitulated in our study among the patients surviving beyond the 18-month landmark and was significantly associated with nBAP1-negative status. Inhibition of ASS1 can reduce proliferation of breast or colorectal cancer (45, 46) and moderately affected BAP1-altered MPM cells expressing ASS1, consistent with the idea that ASS1 can support MPM cell survival (54). In breast cancer, high ASS1 is associated with increased purine synthesis, potentially sensitizing cells to mizoribine (45), an immunosuppressive drug used in renal transplantation; however, in MPM we found no link between mizoribine sensitivity and either ASS1 or BAP1 status. ASS1 inhibition with αMDLA in the presence of extracellular arginine, was not influenced by *BAP1* mutation in MeT5A cells. However, *BAP1*-altered MPM that are more resistant to arginine deprivation with ADI-PEG20, were more sensitive to αMDLA. Thus, these cancer cells may be less able to switch to using extracellular arginine, or have higher arginine demand, than MeT5A cells. Further analysis will be required to dissect out other potential contributory factors, such as extracellular arginine concentration or genetic background, to assess whether ASS1 inhibition could be a useful therapeutic approach in *BAP1*-altered MPM.

While the ADAM and TRAP trials showed promise for ADI-PEG20 in ASS1-deficient epithelioid and nonepithelioid MPM, respectively (6, 55, 56), subsequent clinical development of ADI-PEG20 has focused on patients with nonepithelioid MPM, as high-frequency ASS1 loss in this group avoids the need for screening in patients, reflecting the lack of a validated companion IHC diagnostic for ASS1. The recently announced results of the randomized phase II/III ATOMIC-Meso trial validated this strategy with a 1.64-month median OS benefit [HR = 0.71; 95% confidence interval (CI), 0.55–0.93] in nonepithelioid MPM with combined ADI-PEG20 and cisplatin/pemetrexed compared with standard chemotherapy; supporting imminent regulatory submission (https://polarispharma.com/2022/09/21/20220921001/?lang=en). Meanwhile, the CheckMate-743 study, which initiated concurrently with ATOMIC-Meso and assessed ipilimumab/nivolumab versus standard chemotherapy, reported earlier and led to adoption of ipilimumab/nivolumab as a first-line standard of care (5). Notably, the study showed a striking 9.3-month improvement in OS in the nonepithelioid group (HR = 0.46; 95% CI, 0.31–0.68), while in contrast, the absolute benefit of combination immunotherapy for epithelioid disease was substantially lower at 2.7 months (HR = 0.86; 95% CI, 0.69–1.08), highlighting the need for further treatment options in this group, and supporting reconsideration of ADI-PEG20.

We found that the association between BAP1 status and ASS1 expression was most notable in epithelioid MPM and, as loss of BAP1 expression is associated with resistance to ADI-PEG20, propose that as a validated tool in MPM histopathology, BAP1 IHC will be useful in stratifying epithelioid MPM for ADI-PEG20 treatment. While the benefit of therapeutic stratification by BAP1 status has yet to be realized, our study provides a new avenue for exploration. Further bridging preclinical investigation, beyond the scope of this study, is planned to explore this hypothesis and may reveal additional molecular modulators to further refine predictive models.

## Supplementary Material

Supplementary Methods and References Supplementary Methods, References and List of Figures and Tables

Supplementary Figures S1 to S16 Fig. S1. SNP6.0 analysis of parental MeT5A-BAP1+/+ cells.Fig. S2. Expression of catalytically active BAP1 in parental MeT5A.Fig. S3. Characterisation of BAP1 expression in gene-edited MeT5A.Fig. S4. Whole genome sequencing of gene-edited MeT5A.Fig. S5. Proliferative profiles for gene-edited MeT5A.Fig. S6. Enriched GO terms in gene-edited MeT5A SILAC-MS.Fig. S7. Enriched KEGG pathways in gene-edited MeT5A SILAC-MS include EMT.Fig. S8. Metabolite responses to BAP1 mutation in isogenic MeT5A.Fig. S9. Immunoblotting for differentially expressed metabolic enzymes identified by SILAC-MS in isogenic MeT5A cell lines.Fig. S10. Characterisation of BAP1-status for MPM cell panel.Fig. S11. Evaluating correlation between BAP1 and selected metabolic enzymes in a panel of MPM cell lines and the TCGA MESO pan-cancer dataset.Fig. S12. Validation of ASS1 response to BAP1 alteration.Fig. S13. Improved prognosis for epithelioid MPM patients with loss of nBAP1 and increased expression of ASS1.Fig. S14. Relationship between BAP1 and ASS1 transcripts in the TCGA Pan-Cancer datasets for other cancer types.Fig. S15. The influence of BAP1-status on response to inhibition of purine metabolism.Fig. S16. The influence of BAP1-status on response to ASS1 inhibition.

Supplementary Table S1 STR profiling MPM cell lines

Supplementary Table S2 WGS MeT5A isogenics

Supplementary Table S3 Proteomics MeT5A isogenics and MPM cell lines

Supplementary Table S4 Metabolomics MeT5A isogenics

Supplementary Table S5 Patient scoring and survival summary
