# Supplementary material for: BAP1 Loss Is Associated with Higher ASS1 Expression in Epithelioid Mesothelioma: Implications for Therapeutic Stratification
Source: Mol Cancer Res. 2023 Jan 20;21(5):411–27. doi: 10.1158/1541-7786.MCR-22-0635 (PMC10150242; doi:10.1158/1541-7786.MCR-22-0635)
Supplement: Supplementary Methods and References — Supplementary Methods, References and List of Figures and Tables [file mcr-22-0635_supplementary_methods_and_references_suppsm1.docx]

**Supplementary Material**

**Additional Methods for Supplementary Figures and Tables**

**Copy number analysis**

The genome-wide copy number for the clonal parent line, MeT5A-*BAP1^+/+^*(C2), was determined by SNP Array 6.0 (Affymetrix).

**Cell cycle analysis**

To determine the cell cycle profile of the isogenic MeT5A cell panel, exponentially growing cells were fixed prior to propidium iodide (Sigma-Aldrich) staining to determine DNA content. The fluorescence of single cells was quantified using a BD FACScalibur^TM^ system.

**PCR and qRT-PCR**

Endpoint RT-PCR was performed using Hotstar Taq Master mix (Qiagen) and a Thermo Hybaid PX2; qRT-PCR was performed as described in the methods with normalisation to ACTB and relative expression represented as 2^-[ΔΔCt]^ relative to *BAP1^+/+^* cells. Primer sequences were designed to amplify *BAP1* exons 3 to 4 (For: 5’-GGGTGCAAGTGGAGGAGA-3’, Rev: 5’-GGACGTATCATCCACCAAGG-3’), exons 5/6 to 7 (For: 5’-CAGCCCTGAGAGCAAAGGATATG-3’, Rev: 5’-ATGGTCCGCACTGCACTAAG-3’) and exons 6 to 8 (For: 5’-AGCAAAGGATATGCGATTGG-3’, Rev: 5’-GATACGCTCCATGATGACC-3’), or to amplify between the PGK promoter and the Neomycin gene of the promoter trap (For: 5’-GTCTGGAGCATGCGCTTTAG-3’, Rev: 5’-TAGCCGAATAGCCTCTCCAC-3’). For qRT-PCR fibronectin (FN1) primers were For: 5’-CCTGACAGCTCATCCGTGG-3’, Rev: 5’-TCAGTAGCATCTGTCACACG-3’.

**Ubiquitin active site-directed probe assay**

Cells were lysed in non-denaturing buffer (50mM Tris pH7.5, 5mM MgCl_2_, 250mM sucrose, 1mM DTT, 2mM ATP) on ice and homogenized by progressively passing through 23G, 26G and 30G needles, PhosStop (Roche) was immediately added. Lysates were cleared by centrifugation (20 min, 14,000 rpm, 4°C) and a BCA assay performed to standardize protein concentrations. 15μg protein was incubated at 37°C with shaking at 300rpm with HA-Ub-VME (UbiQ-035, UbiQ,) or HA-Ub-PA (UbiQ-078) giving a 1:100 or 1:200 ratio of probe:protein and incubated as indicated in the figure legends. Sample buffer (5X: 15% SDS, 312.5mM Tris pH6.8, 50% glycerol, 16% β-mercaptoethanol, bromophenol blue) was added and reactions terminated at 95°C for 5 min before immunoblotting for BAP1.

**Whole genome sequencing of MeT5A cell lines**

Genomic DNA was extracted from MeT5A isogenic cell lines using a DNeasy Blood and Tissue Kit (Qiagen, Hilden, Germany) and sent to Novogene (Cambridge Science Park, UK) for quality control, library preparation, sequencing and bioinformatic analysis. Genomic DNA was randomly sheared, end repaired and A-tailed, ligated with an Illumina adapter, PCR amplified, size selected, and purified. The library was checked using Qubit and real-time PCR for quantification and a bioanalyzer for size distribution, before being pooled and sequenced on an Illumina Novaseq 6000 system. Data were quality controlled and paired-end reads discarded that contained adapter contamination, >10% uncertain bases, or >50% low-quality bases (Phred <5). Clean reads were >99.85%, Phred scaled quality scores above 30 were >89.2% and error rates were 0.03%. Burrows-Wheeler Aligner (BWA; RRID:SCR_010910) was used to map paired-end clean reads to the human reference genome (hg38, <http://hgdownload.cse.ucsc.edu/goldenPath/hg38/bigZips/analysisSet/hg38.analysisSet.2bit>. Minimum statistics across the samples were >99.0% paired-end reads properly mapped, average sequencing depth >31.7X, and >84.1% coverage at >20X. Mutation detection included single nucleotide polymorphisms (SNPs), small insertions and deletions (InDels), structural variants (SVs) and copy number variants (CNVs). The tools muTect and Strelka were used to detect somatic SNPs and InDels, respectively. Following genomic variant detection, annotation was performed using ANNOVAR (Suppl. ref. 1), including to identify protein coding changes and affected genomic regions (RefSeq and Gencode), predict deleteriousness (SIFT, PolyPhen, MutationAssessor, LRT and CADD) or conservation (GERP++ scores) of mutations, and provide functional or pathway annotation (Gene Ontology, KEGG, Reactome, Biocarta and PID). Annotated data provided by Novogene were interrogated using Gigasheet to filter genomic variants according to functional annotations and cancer relevance, as indicated in supplementary table 2.

**Bioinformatics**

Functional analysis and visualisation of proteomics data from gene-edited MeT5A *BAP1^w-/KO^* clone C5.1 and the parental MeT5A *BAP1^+/+^* cell line was performed using the ProteoRE (Proteomics Research Environment) Galaxy-based platform (Suppl. ref. 2) available at <https://proteore.org>. Enrichment of GO terms amongst differential proteins with >1.5-fold change was calculated against the background of all proteins identified in the MS experiment, using Fisher’s exact test with Benjamini-Hochberg correction. TCGA Pan-Cancer datasets (Suppl. ref. 3) were analysed using cBioportal or exported for local analysis.

**Immunoblotting antibodies**

Immunoblotting was performed as described in the methods using additional primary antibodies: mouse anti-pan-cytokeratin (sc8018, Santa Cruz, RRID:AB_627396), anti-SERPINE1 (ab82218; Abcam, RRID:AB_1658476) and rabbit anti-FN1 (F3648; Sigma Merck, RRID:AB_476976), anti-mesothelin (D4X7M, Cell Signaling, RRID:AB_2799681), anti-P16 (sc-468, Santa Cruz, RRID:AB_632103), anti-NF2 (ab109244, Abcam, RRID:AB_10866511), anti-ASS1 (HPA020934; Sigma Merck, RRID:AB_1845118) or anti-BAP1 (D1W9B, #13187 Cell Signaling, RRID:AB_2798143) for N-terminal BAP1 detection.

**Immunofluorescence**

Cells were fixed as previously described (36) and incubated with primary then secondary antibodies at room temperature for 1 hour in 10% goat serum: mouse BAP1 (sc-28383, Santa Cruz, RRID:AB_626723), rabbit B-tubulin (ab6046, Abcam, RRID:AB_2210370), anti-mouse Alexa-Flour488 and anti-rabbit Alexa-Flour594 (Molecular Probes). Coverslips were mounted onto slides in Moviol containing DAPI (D1306, Invitrogen). Cells were imaged using a Nikon Eclipse Ti (CFI Super Plan Fluor ELWD ADM 20XC N.A. 0.45).

**BAP1 transcript sequencing**

RNA extraction (with on-column DNAse treatement) and cDNA conversion we performed as described in the methods. Endpoint PCR was performed with Q5 High-Fidelity DNA polymerase (New England Biolabs) and primers designed to amplify the full coding sequence (For: 5’-ATGAATAAGGGCTGGCTGGA-3’, Rev: 5’-GCTGCGTGTTGACTGCATAC-3’). Amplicons were separated on 1% agarose gels and purified using the Wizard® SV Gel and PCR Clean-Up System (Promega). Sequencing was performed by Dundee Sequencing Services with primers covering the full BAP1 transcript: 5’-CAGCCCTGAGAGCAAAGGATATG-3’; 5’-ATGGTCCGCACTGCACTAAG-3’; 5’-ACAAGGCCAAGCGCCAGTGA-3’; 5’-TAGTGGTGAAGCCTCCAG-3’; 5’-GCTGCGTGTTGACTGCATAC-3’.

**RNA Interference**

MeT5A cells were seeded 24 hours prior to transfection with 10nM siRNA using Oligofectamine (Invitrogen) then analysed 72 h later by immunoblotting. ASS1 was depleted with siRNAs (Qiagen): siASS#2 (SI00000805), siASS1#3 (SI04434248), siASS1#4 (SI04434255), siASS1#5 (SI05133842) or siASS1#6 (SI05133849), together with the All-Stars negative control siRNA (1027281, siC) or mock transfection.

**Statistical analyses of patient data**

Continuous data are summarised as median (IQR) and categorical data are summarised as frequencies of counts with associated percentages. The outcome is overall survival measures as the time from diagnosis until death by any cause. Of primary interest are the prognostic impact of ASS1, measured as a continuous covariate and BAP1 measured as a binary factor. Survival estimates are obtained using the method of Kaplan and Meier with Cox proportional hazards models to evaluate the prognostic impact of ASS1 and BAP1 while adjusting for patient age and gender. Upon observation of non-proportional hazards caused by delayed effects of the biomarkers, landmark analyses were performed using landmarks of 12, 18 and 24 months. Results are presented in terms of hazard ratios (95% Confidence intervals) with a *P*-value of 0.05 used to determine statistical significance. All analyses are performed using R (Version 4).

**Supplementary References**

**Suppl. ref 1.** Wang K, Li M, Hakonarson H. ANNOVAR: Functional annotation of genetic variants from high-throughput sequencing data. Nucleic Acids Res 2010, 38(16): e164.

**Suppl. ref 2.** Combes F, Loux V, Vandenbrouck Y. GO Enrichment Analysis for Differential Proteomics Using ProteoRE. Methods Mol Biol 2021;2361:179-196.

**Suppl. ref 3.** Hoadley KA, Yau C, Hinoue T, Wolf DM, Lazar AJ, Drill E*, et al.* Cell-of-Origin Patterns Dominate the Molecular Classification of 10,000 Tumors from 33 Types of Cancer. Cell 2018;173:291-304 e6.

**Suppl. ref 4.** Yoshikawa Y, Sato A, Tsujimura T, Emi M, Morinaga T, Fukuoka K, et al. Frequent inactivation of the *BAP1* gene in epithelioid-type malignant mesothelioma. Cancer Sci 2012;103:868-74.

**Suppl. ref 5.** Bepler G, Koehler A, Kiefer P, Havemann K, Beisenherz K, Jaques G et al. Characterization of the state of differentiation of six newly established human non-small-cell lung cancer cell lines. Differentiation 1988; 37: 158-171.

**Suppl. ref 6.** Phelps RM, Johnson BE, Ihde DC, Gazdar AF, Carbone DP, McClintock PR et al. NCI-Navy Medical Oncology Branch cell line data base. J Cell Biochem Suppl 1996; 24: 32-91.

**Supplementary Figures list** (see PDF)

Supplementary Figure S1. SNP6.0 analysis of parental MeT5A-*BAP1^+/+^* cells.

Supplementary Figure S2. Expression of catalytically active BAP1 in parental MeT5A.

Supplementary Figure S3. Characterisation of BAP1 expression in gene-edited MeT5A.

Supplementary Figure S4. Whole genome sequencing of gene-edited MeT5A.

Supplementary Figure S5. Proliferative profiles for gene-edited MeT5A.

Supplementary Figure S6. Enriched GO terms in gene-edited MeT5A SILAC-MS.

Supplementary Figure S7. Enriched KEGG pathways in gene-edited MeT5A SILAC-MS include EMT.

Supplementary Figure S8. Metabolite responses to *BAP1* mutation in isogenic MeT5A.

Supplementary Figure S9. Immunoblotting for differentially expressed metabolic enzymes identified by SILAC-MS in isogenic MeT5A cell lines.

Supplementary Figure S10. Characterisation of BAP1-status for MPM cell panel.

Supplementary Figure 11. Evaluating correlation between BAP1 and selected metabolic enzymes in a panel of MPM cell lines and the TCGA MESO pan-cancer dataset.

Supplementary Figure 12. Validation of ASS1 response to BAP1 alteration.

Supplementary Figure 13. Improved prognosis for epithelioid MPM patients with loss of nBAP1 and increased expression of ASS1.

Supplementary Figure 14. Relationship between *BAP1* and *ASS1* transcripts in the TCGA Pan-Cancer datasets for other cancer types.

Supplementary Figure 15. The influence of BAP1-status on response to inhibition of purine metabolism.

Supplementary Figure 16. The influence of BAP1-status on response to ASS1 inhibition.

**Supplementary Tables list** (see Excel files)

Suppl Table 1 – STR profiling MPM cell lines

Suppl Table 2 – WGS MeT5A isogenics

Suppl Table 3 – Proteomics MeT5A isogenics and MPM cell lines

Suppl Table 4 – Metabolomics MeT5A isogenics

Suppl Table 5 – Patient scoring and survival summary
