# Supplementary material for: BAP1 Loss Is Associated with Higher ASS1 Expression in Epithelioid Mesothelioma: Implications for Therapeutic Stratification
Source: Mol Cancer Res. 2023 Jan 20;21(5):411–27. doi: 10.1158/1541-7786.MCR-22-0635 (PMC10150242; doi:10.1158/1541-7786.MCR-22-0635)
Supplement: Supplementary Figures S1 to S16 — Fig. S1. SNP6.0 analysis of parental MeT5A-BAP1+/+ cells.Fig. S2. Expression of catalytically active BAP1 in parental MeT5A.Fig. S3. Characterisation of BAP1 expression in gene-edited MeT5A.Fig. S4. Whole genome sequencing of gene-edited MeT5A.Fig. S5. Proliferative profiles for gene-edited MeT5A.Fig. S6. Enriched GO terms in gene-edited MeT5A SILAC-MS.Fig. S7. Enriched KEGG pathways in gene-edited MeT5A SILAC-MS include EMT.Fig. S8. Metabolite responses to BAP1 mutation in isogenic MeT5A.Fig. S9. Immunoblotting for differentially expressed metabolic enzymes identified by SILAC-MS in isogenic MeT5A cell lines.Fig. S10. Characterisation of BAP1-status for MPM cell panel.Fig. S11. Evaluating correlation between BAP1 and selected metabolic enzymes in a panel of MPM cell lines and the TCGA MESO pan-cancer dataset.Fig. S12. Validation of ASS1 response to BAP1 alteration.Fig. S13. Improved prognosis for epithelioid MPM patients with loss of nBAP1 and increased expression of ASS1.Fig. S14. Relationship between BAP1 and ASS1 transcripts in the TCGA Pan-Cancer datasets for other cancer types.Fig. S15. The influence of BAP1-status on response to inhibition of purine metabolism.Fig. S16. The influence of BAP1-status on response to ASS1 inhibition. [file mcr-22-0635_supplementary_figures_s1_to_s16_suppsf1-16.pdf]

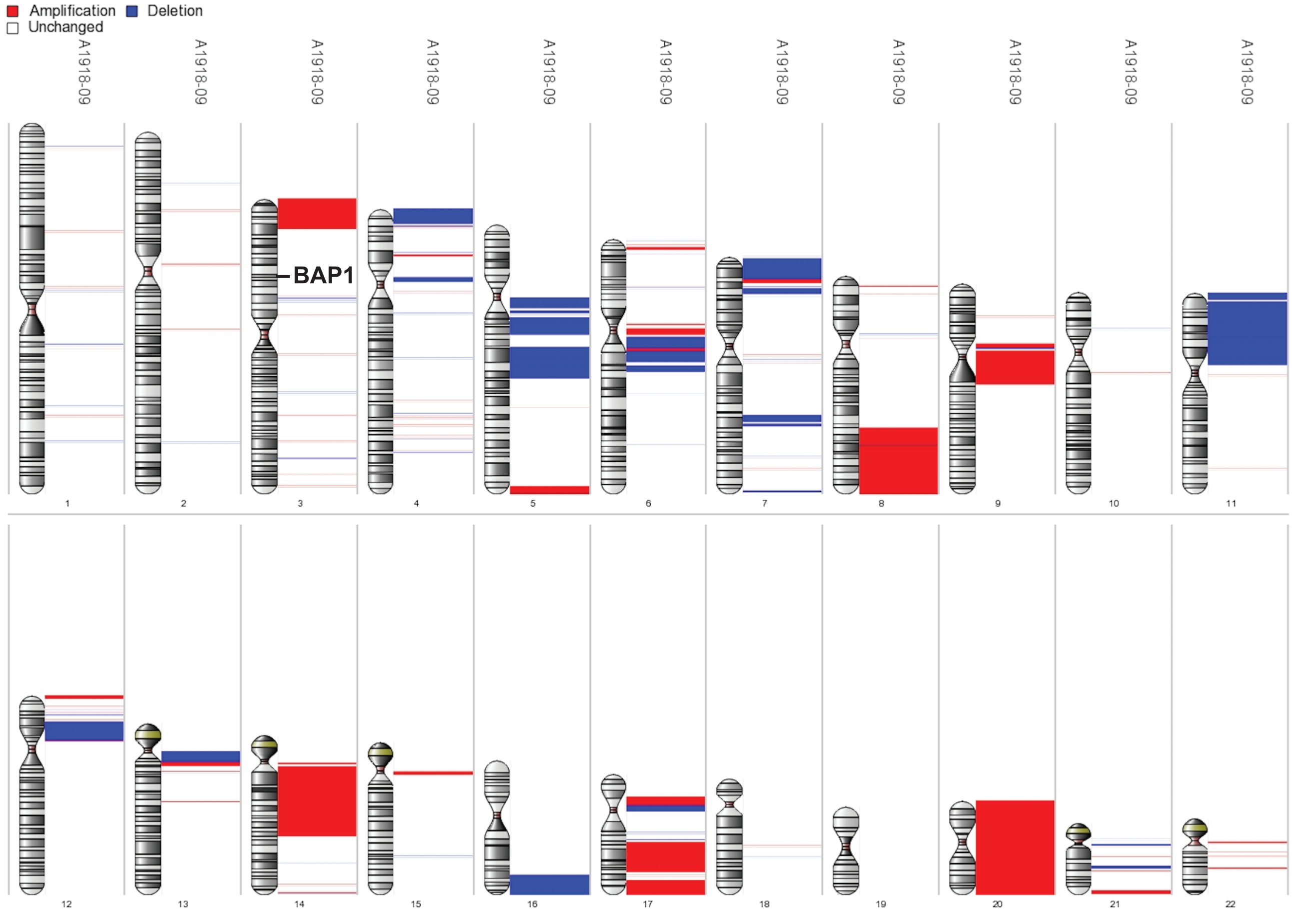

Supplementary Figure S1. SNP6.0 analysis of parental MeT5A-*BAP1*$^{+/+}$ cells. The clonal parental cells are mainly diploid with some regions of amplification (red) or deletion (blue). Importantly, they are diploid around the *BAP1* locus at 3p21.

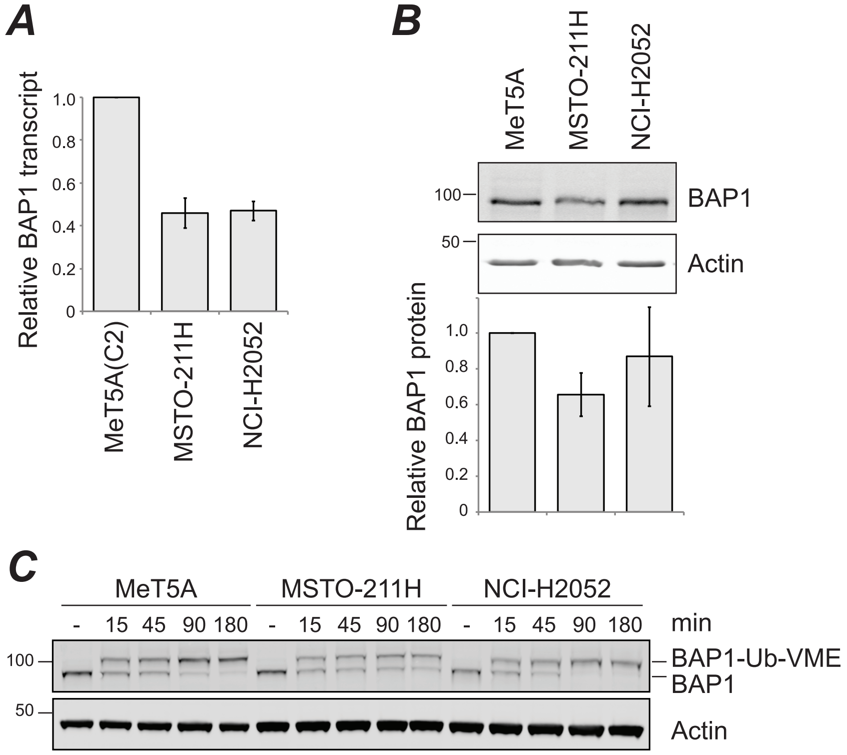

Supplementary Figure S2. Expression of catalytically active BAP1 in parental MeT5A. Comparison of the clonal parental MeT5A-*BAP1*$^{+/+}$ line and two MPM cell lines with verified wild-type *BAP1* (15) (Suppl. ref 4): MSTO-211H (biphasic, from pleural effusion) (Suppl. ref 5) and NCI-H2052 (sarcomatoid, from a metastatic lymph node) (Suppl. ref 6). **A,** qRT-PCR analysis of BAP1 mRNA expression (exons 3-to-4) normalised to actin; mean values from 3 independent experiments; error bars SD. **B,** Representative immunoblot (top) and quantification (below) showing mean values normalised to actin from 3 independent experiments; error bars SD. **C,** Ub-VME probe binding assay to demonstrate BAP1 catalytic activity. Non-denaturing cell lysates (15μg protein) were incubated with HA-Ub-VME as indicated at 1:200 probe:protein. Endogenous BAP1 protein bound to the active site-directed probe ubiquitin-VME results in a 10kDa mobility shift. BAP1 reactivity is reduced in MSTO-211H relative to MeT5A.

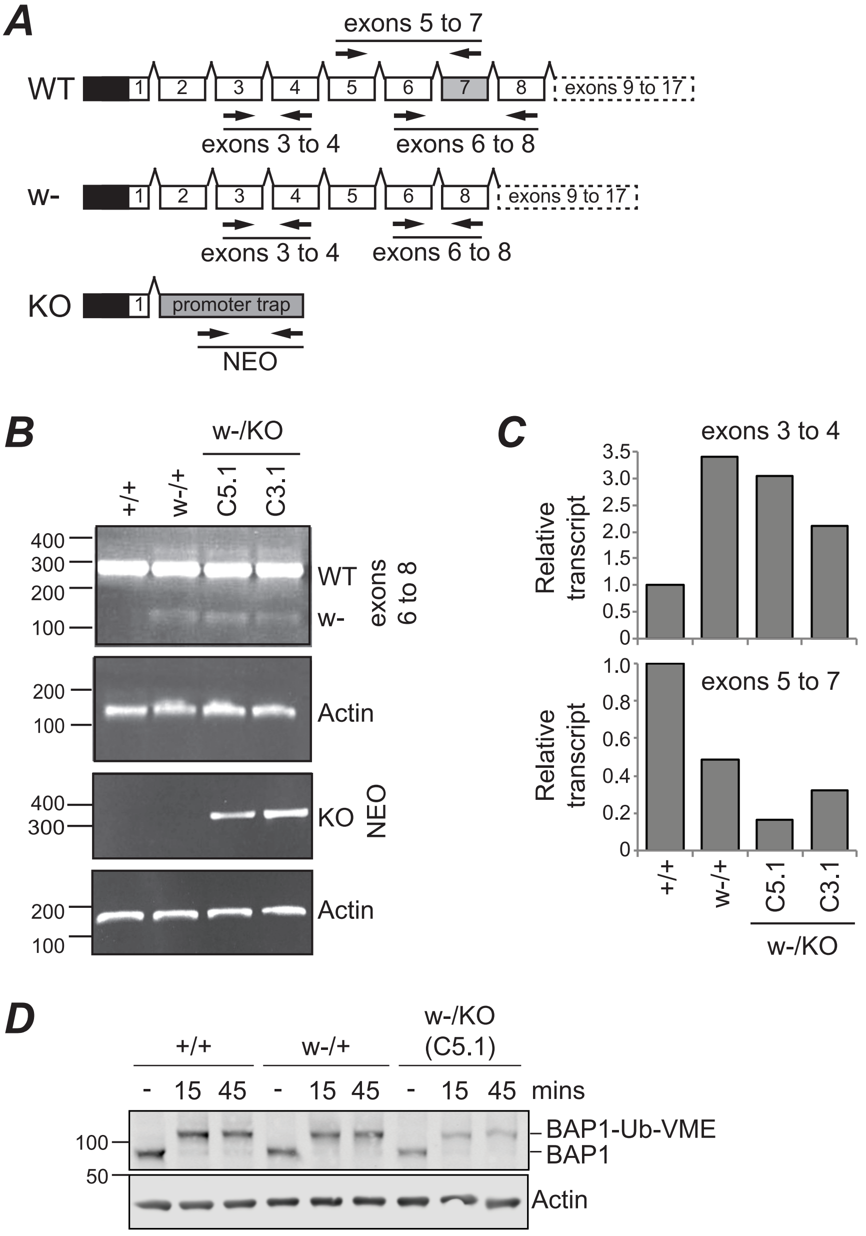

Supplementary Figure S3. Characterisation of BAP1 expression in gene-edited MeT5A.
A, Schematic of RT-PCR primers used to characterise transcription, illustrating amplicons for wild type (WT) alleles or after successful w- or KO genome-editing. **B,** End-point RT-PCR across exons 6-to-8 shows WT transcript (258bp) and the w-allele splice variant (115bp) and across the NEO transcript sequence confirms the presence of the promoter trap in *BAP1$^{w-/KO}$* clones. **C,** qRT-PCR shows that the w-mutation increases overall BAP1 locus transcription (exons 3-to-4) but confirms reduced WT transcript expression (exons 5-to-7); some WT transcript is detected in *BAP1$^{w-/KO}$* through incomplete mis-splicing of the w-allele. **D,** Residual BAP1 in gene-edited MeT5A cells is catalytically active. Non-denaturing cell lysates (15μg protein) were incubated with HA-Ub-VME as indicated at 1:200 probe:protein.

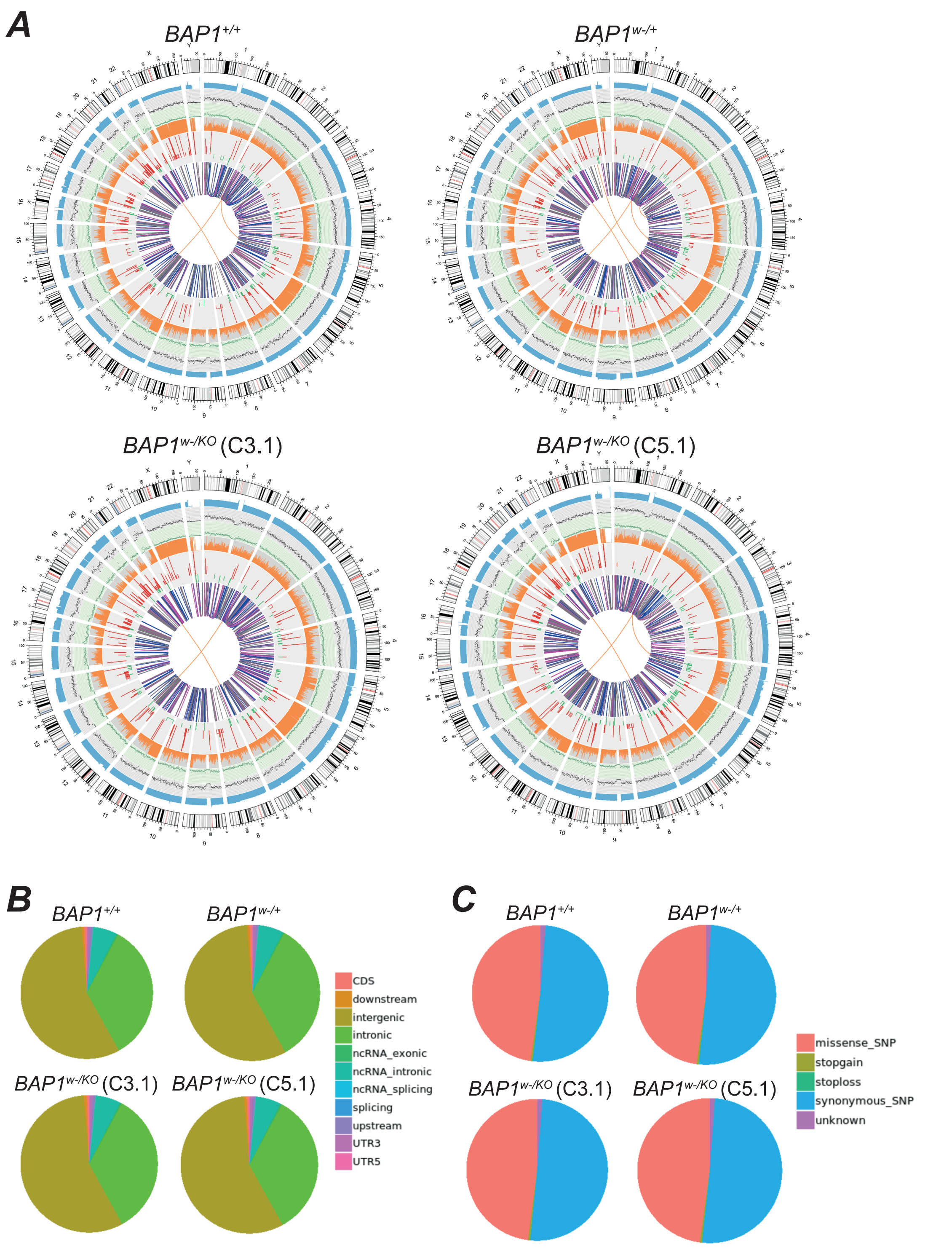

Supplementary Figure S4. Whole genome sequencing (WGS) of gene-edited MeT5A. WGS was compared for the parental MeT5A-*BAP1*$^{+/+}$ cells, the haplo-insufficient *BAP1*$^{w-/+}$ clone and the two double-mutant *BAP1*$^{w-/KO}$ clones C3.1 and C5.1. **A,** Circo plots for each cell line summarising from the outside ring inwards: chromosome information; read coverage (blue bars, 0.5Mbp); InDel density (black dots, 1Mbp); SNP density (green dots, 1Mbp); proportion homozygous SNP (orange bars, 1Mbp) or heterozygous SNP (grey); CNV inference (gain red, loss green); SV inference in exonic and splicing regions (BND orange, INS green, DEL grey, DUP pink, INV blue). **B & C,** Number of SNPs in different genomic regions (**B**) and number of different types of SNPs within coding regions (**C**).

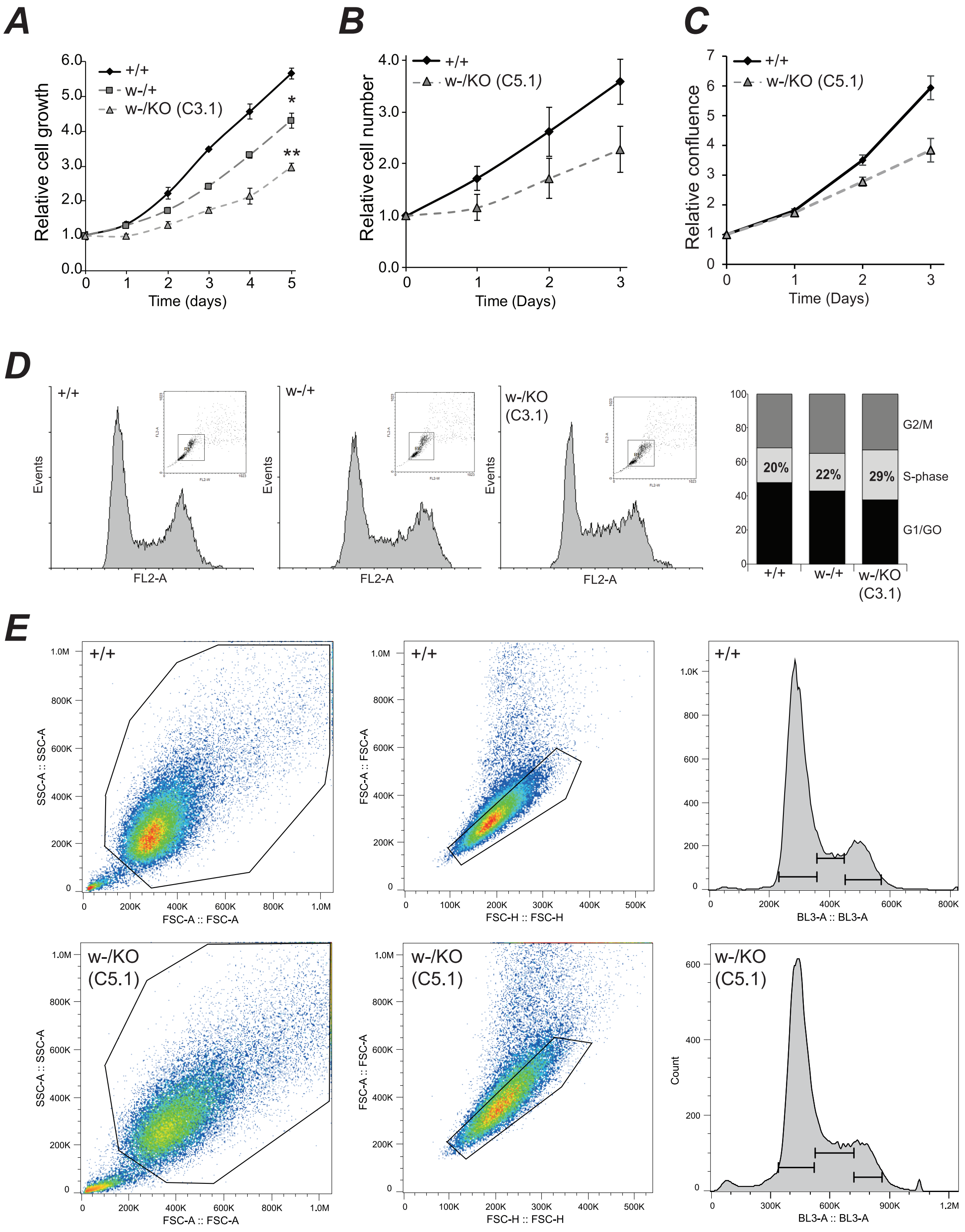

Supplementary Figure S5. Proliferative profiles for gene-edited MeT5A.
A-C, BAP1-deficiency slows proliferation of MeT5A cells. **A,** ATP-based luciferase assay for MeT5A-*BAP1*$^{w-/+}$ and *BAP1*$^{w-/KO}$ clone C3.1; mean data from 3 independent experiments, error bars SD, *P<0.05 or **P<0.01 compared to MeT5A-*BAP1*$^{+/+}$ by one-way ANOVA and Dunnett post-hoc test. **B & C,** Proliferation of *BAP1*$^{w-/KO}$ C5.1 was assessed by direct cell counts (**B**) and live imaging of cell confluence (**C**). **D & E,** BAP1-deficiency causes MeT5A cells to accumulate in S-phase. Representative flow cytometry for MeT5A-*BAP1*$^{w-/+}$ and *BAP1*$^{w-/KO}$ clone C3.1 (**D**) and supporting data for *BAP1*$^{w-/KO}$ clone C5.1 (**E**).

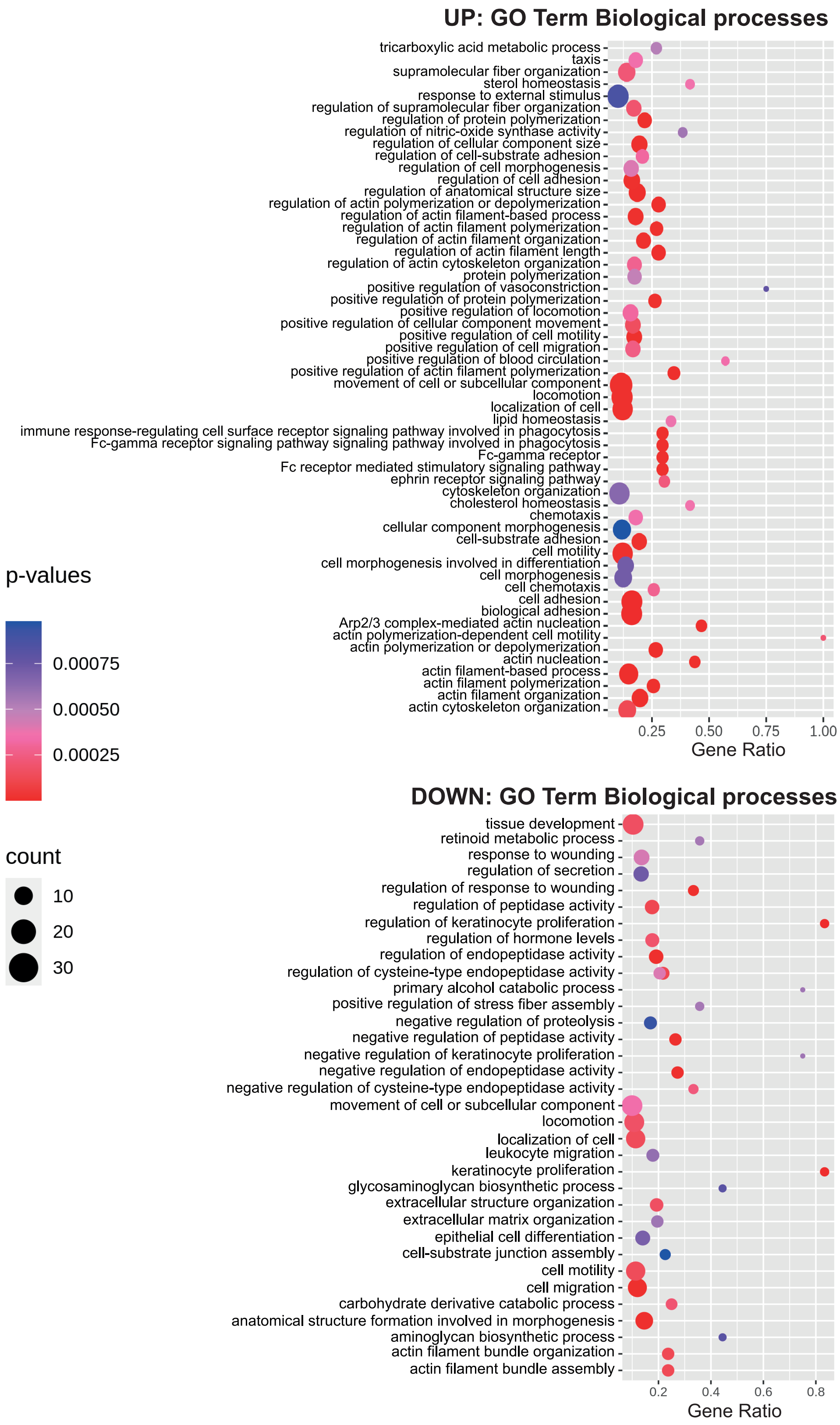

Supplementary Figure S6. Enriched GO terms in gene-edited MeT5A SILAC-MS. Proteins with differential expression of >1.5-fold between MeT5A-*BAP1*$^{w-/KO}$ C5.1 and parental *BAP1*$^{+/+}$ was analysed and visualised separately for the upregulated or down-regulated proteins using ProteoRE. Enrichment of Biological Process GO Terms is shown relative to all proteins identified in the SILAC-MS experiments, using Fisher's exact test with Benjamini-Hochberg correction. Significance of pathway enrichment is indicated by the colour scale, enrichment on the x-axis, and number of differentially expressed proteins within the enriched pathway by bubble size (count).

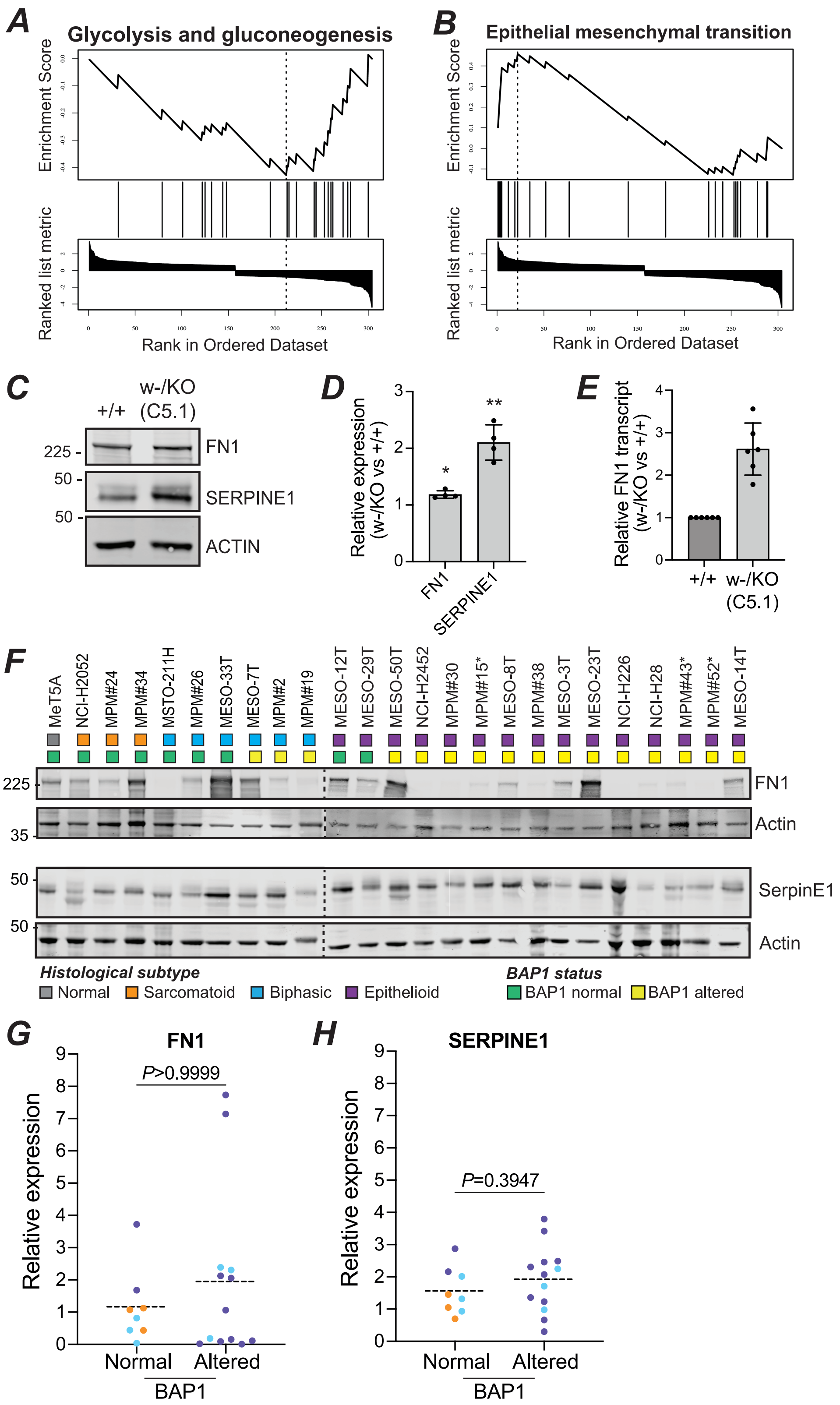

Supplementary Figure S7. Enriched KEGG pathways in gene-edited MeT5A SILAC-MS include EMT.
A-B, Gene Set Enrichment Analysis of KEGG pathways highlights glycolysis/gluconeogenesis (**A**) and epithelial mesenchymal transition (**B**) in gene-edited MeT5A. **C-E,** Selected EMT markers are increased in *BAP1*$^{w-/KO}$ C5.1 cells. Representative immunoblot (**C**) and protein expression relative to *BAP1*$^{+/+}$ MeT5A (**D**); mean of 4 independent experiments, error bars SD, one sample t-test *P= 0.0118, **P=0.0058. Relative FN1 mRNA expression (**E**); mean of 6 independent experiments, error bars SD, one sample t-test **P=0.0013. **F-H,** FN1 and SERPINE protein levels do not stratify by BAP1 -status in an MPM cell panel. Representative immunoblot (**F**) and BAP1-stratified protein expression for FN1 (**G**) and SERPINE1 (**H**); bar indicates mean, statistical analyses unpaired t-test. Key indicates histological subtype and BAP1-status. Note: *MPM#15, MPM#43 and MPM#52 cell lines were excluded from the study after issues revealed by STR profiling. Supporting data, Supplementary Fig. S10.

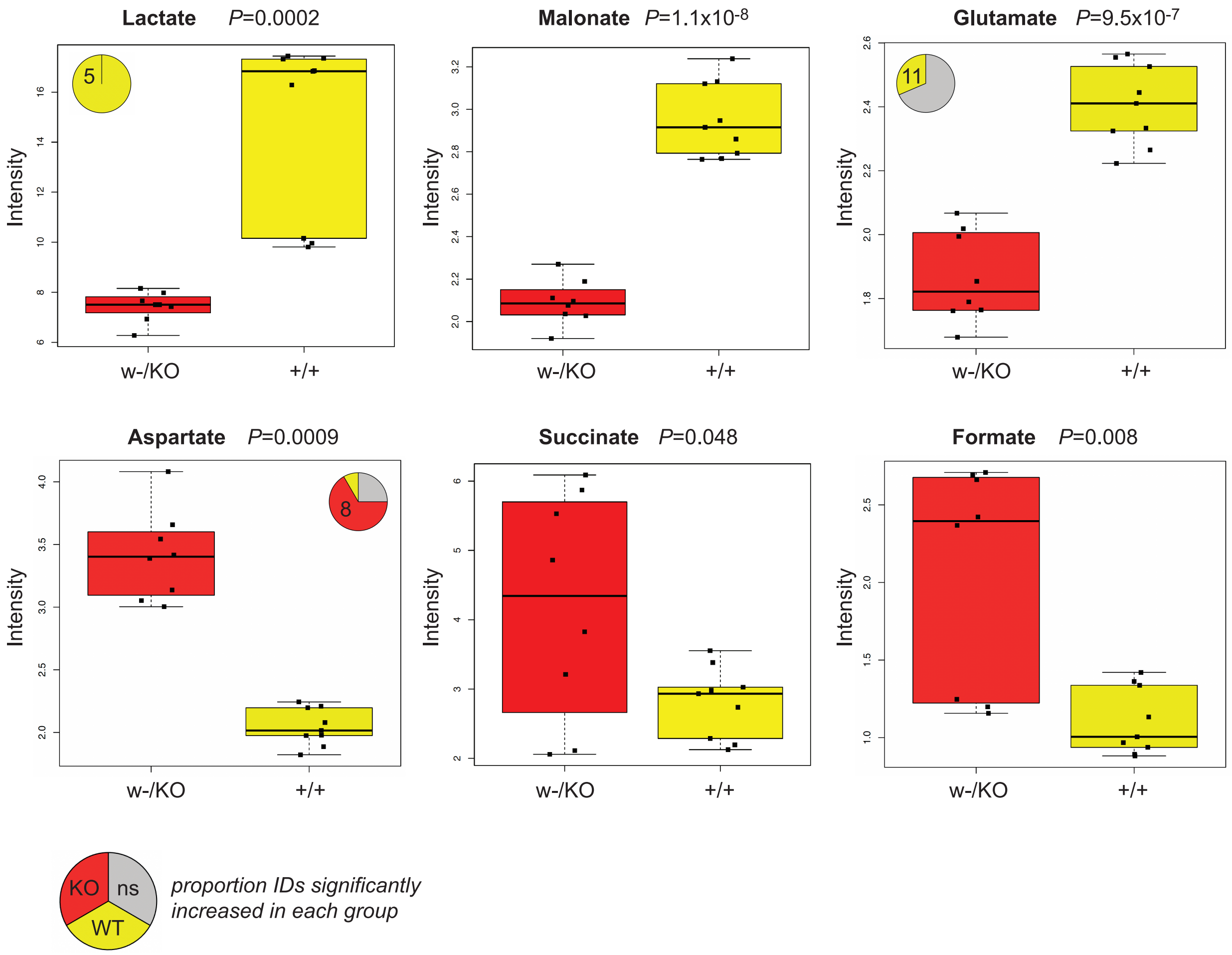

Supplementary Figure S8. Metabolite responses to BAP1 mutation in isogenic MeT5A. Box plots for named metabolites identified by NMR that were significantly modulated in MeT5A-*BAP1*$^{w\text{-}/KO}$ C5.1 relative to *BAP1*$^{+/+}$ cells. Data represent 3 technical replicates for 3 independent extracts of each cell line. Statistical analysis by t-test. Where metabolites were identified more than once, pie charts summarise the proportion of IDs with significant upregulation in *BAP1*$^{w\text{-}/KO}$ C5.1 or *BAP1*$^{+/+}$ MeT5A, or no significant difference (ns).

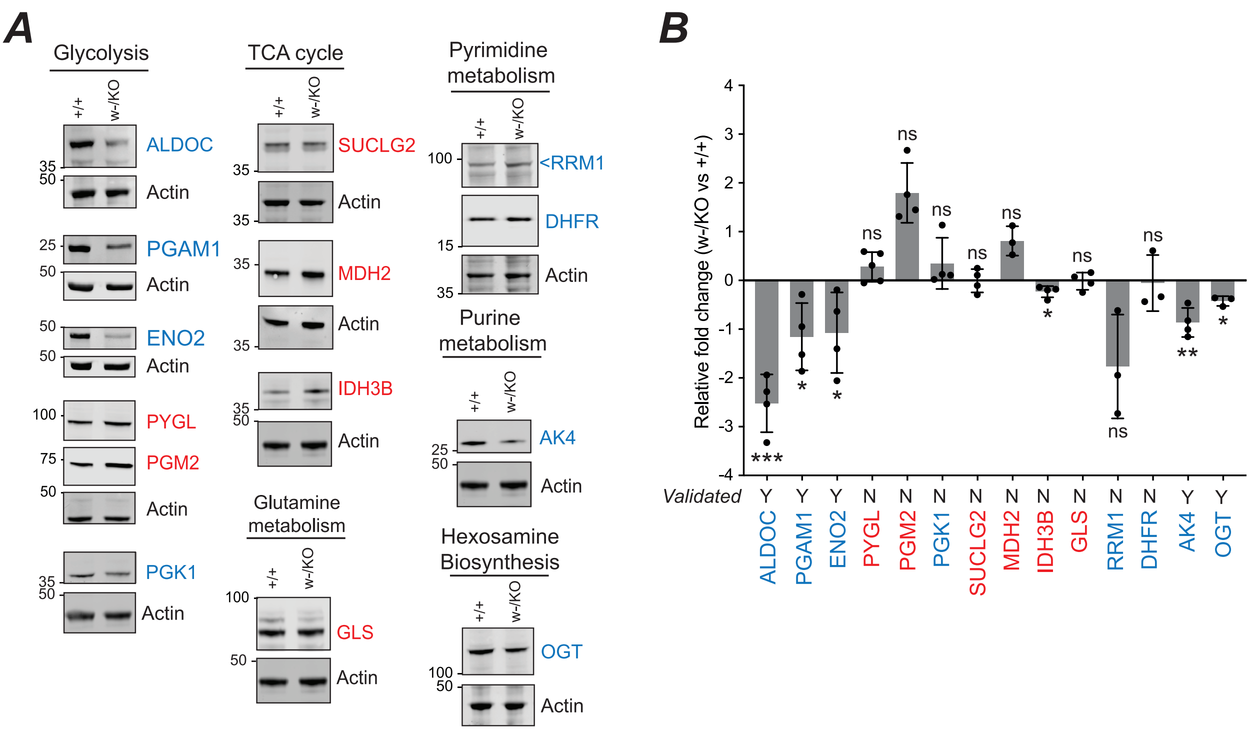

Supplementary Figure S9. Immunoblotting for differentially expressed metabolic enzymes identified by SILAC-MS in isogenic MeT5A cell lines.
Representative blots (**A**) and mean values (**B**) for 3 independent lysates from unlabelled MeT5A cells; error bars SD, one sample t-test on non-log transformed data. Colour coding indicates proteins that were up-regulated (red) or down-regulated (blue) by SILAC-MS in MeT5A-*BAP1*$^{w-/KO}$ C5.1 relative to *BAP1*$^{+/+}$ cells. Responses identified by MS and validated by immunoblotting are indicated in panel **B.**

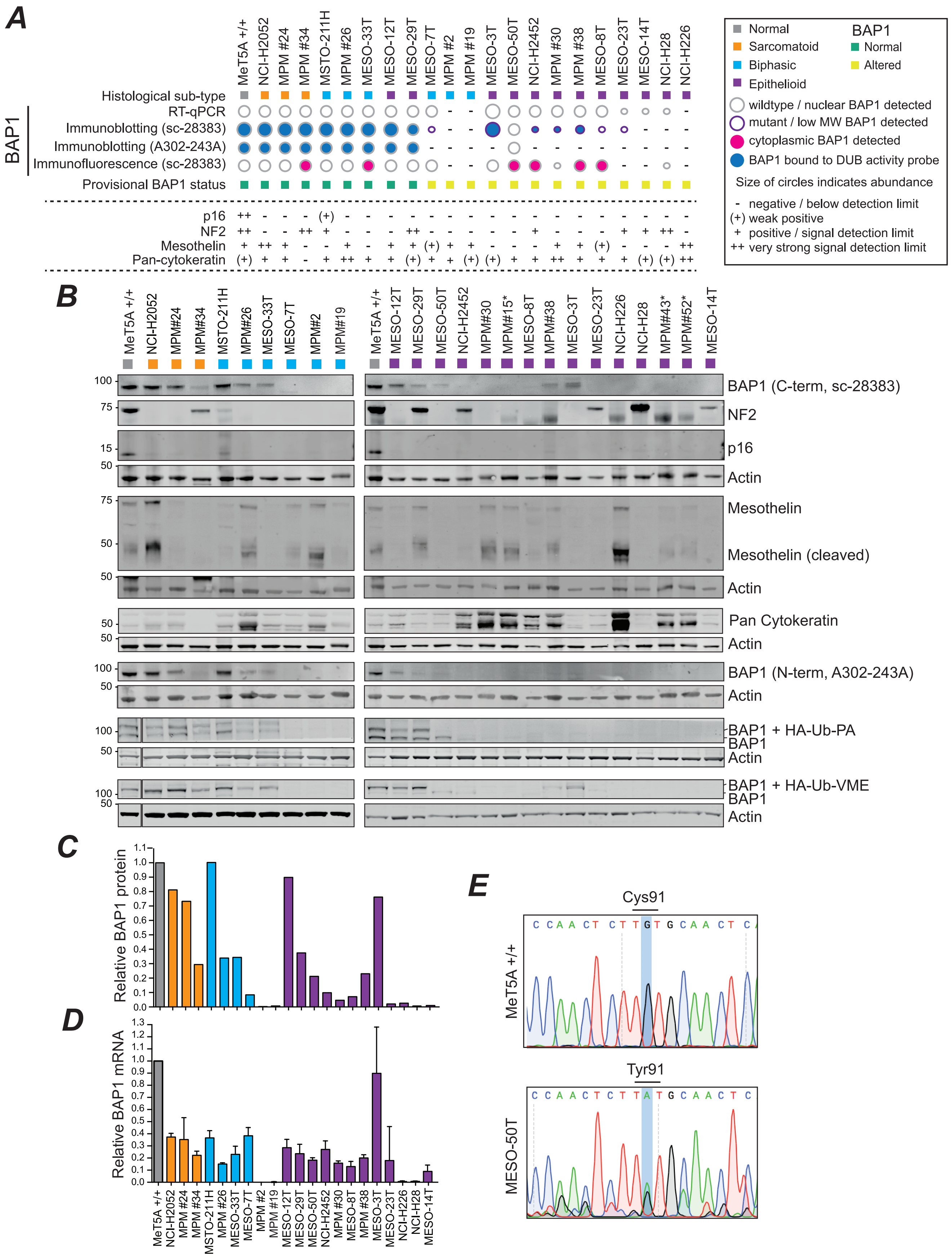

Supplementary Figure S10. Characterisation of BAP1-status for MPM cell panel.
A, Quilt map summarizing characterisation of MPM cell line. Provisional BAP1 status determined by multiple methods as summarised in key, and expression of other MPM markers screened; supporting data in subsequent panels. **B-C,** Immunoblotting for BAP1 and other MPM markers. Representative blots (**B**) and quantification of BAP1 (**C**). Interaction of catalytic competent BAP1 with HA-Ub-PA or -VME produces 10kDa shift in BAP1 immunoreactive band; ubiquitin activity probes incubated with non-denaturing cell lysates (15μg protein, 45 min,1:100 probe:protein). **D,** Mean BAP1 mRNA expression determined by qRT-PCR; normalised to actin relative to MeT5A-*BAP1*$^{+/+}$ from 3 independent experiments, error bars SD. **E,** BAP1 in MESO-50T did not react with the ubiquitin activity probes (panel B) and sequencing of *BAP1* identified a G to A point substitution at CDS 272 (NM_004656) that converts the catalytic cysteine to a tyrosine (C91Y), a somatic mutation that has been reported in both MPM and renal clear cell carcinoma tissues (COSMIC). Note: *MPM#15, MPM#43 and MPM#52 cell lines were excluded from the study as issues revealed by STR profiling.

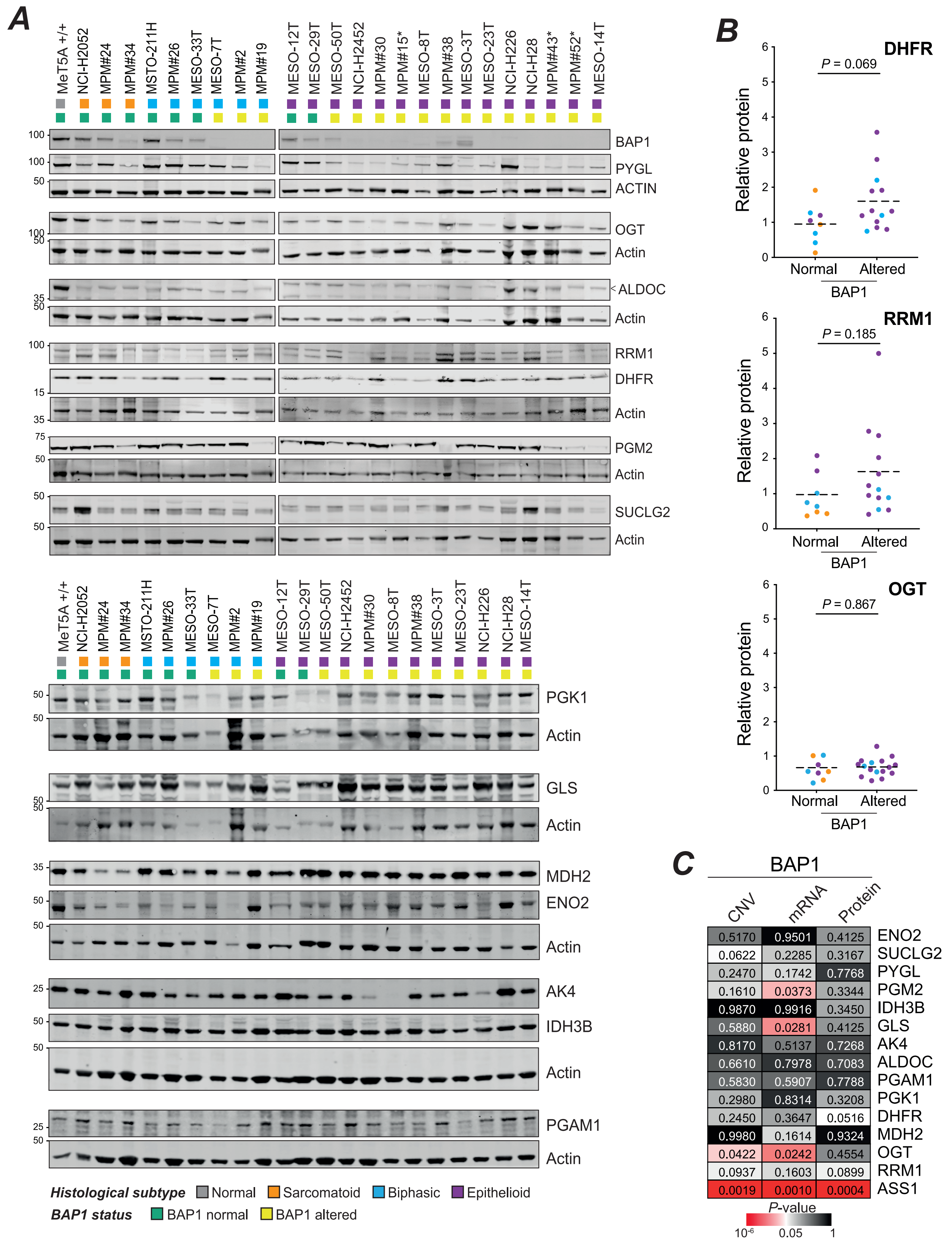

Supplementary Figure S11. Evaluating correlation between BAP1 and selected metabolic enzymes in a panel of MPM cell lines and the TCGA MESO pan-cancer dataset.

A-B, Immunoblotting of MPM cell lines for metabolic enzymes identified by SILAC-MS in isogenic MeT5A cells. Representative blots (**A**) and plots illustrating BAP1-stratification for selected enzymes (**B**). Statistical analyses: DHFR & OGT, unpaired t-test; RRM1 Mann-Whitney. Key indicates histological subtype and BAP1-status. Note: *MPM#15, MPM#43 and MPM#52 cell lines were excluded from the study as issues revealed by STR profiling.

C, ASS1 correlates most strongly with BAP1-status in the TCGA MESO cohort. Heatmap of Spearman correlation *P*-values between mRNA expression for metabolic enzymes of interest and ++ copy number variation (CNV; n=87), mRNA (n=87) and protein (RPPA, n=63) within the TGCA pan-cancer dataset for all MPM cases. The results in **C** are based upon data generated by the TCGA Research Network: https://www.cancer.gov/tcga.

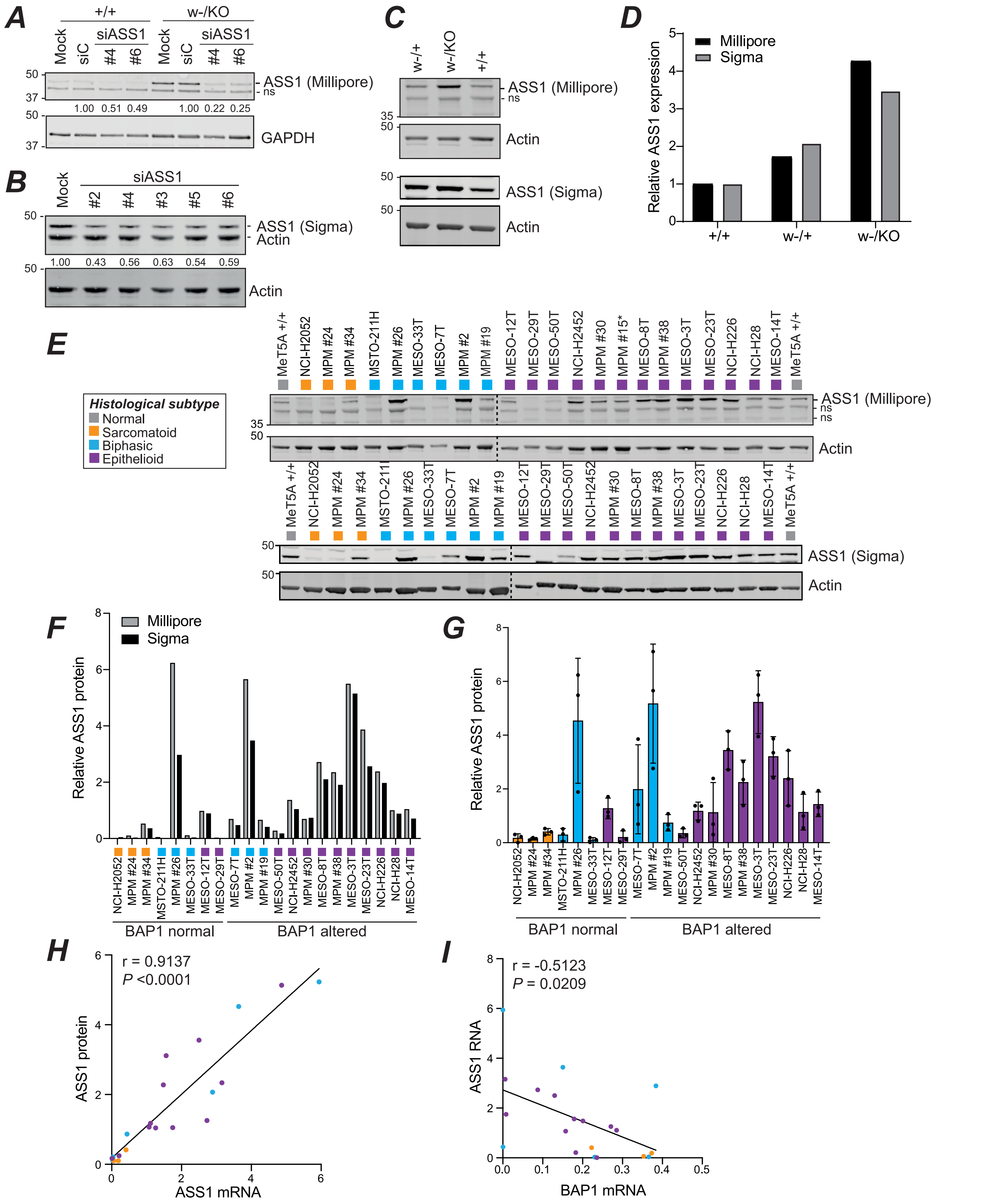

Supplementary Figure S12. Validation of ASS1 response to BAP1 alteration.
A-B, Validation of antibodies for immunoblotting by ASS1 knockdown in isogenic MeT5A cell lines: mouse anti-ASS1 (MABN704, Millipore; immunoblots Fig. 4) (**A**) and rabbit anti-ASS1 (HPA020934; Sigma Merck; IHC Fig. 5) (**B**). **C-D,** ASS1 protein levels increase with sequential BAP1 mutation in isogenic MeT5A cells, by immunoblotting with either antibody. Representative blots (**C**) and quantification (n=1, **D**). **E-G,** Immunoblotting for ASS1 protein levels using either ASS1 antibody confirms higher expression in BAP1-altered MPM cell lines. Representative blots (**E**), comparison of quantification for the two antibodies from these blots (**F**), and mean values for detection of ASS1 (Millipore antibody) from 3 independent experiments, error bars SD, corresponding with data in Figure 4F-4H (**G**). Colour coding indicates histological subtypes. Note: *MPM#15 cell line excluded from study as issues revealed on STR profiling. **H,** ASS1 mRNA and protein expression (Millipore antibody) are positively correlated across the MPM cell panel. **I,** BAP1 and ASS1 mRNA expression are inversely correlated for the MPM cell panel. For F-I, histological subtype is indicated in panel E.

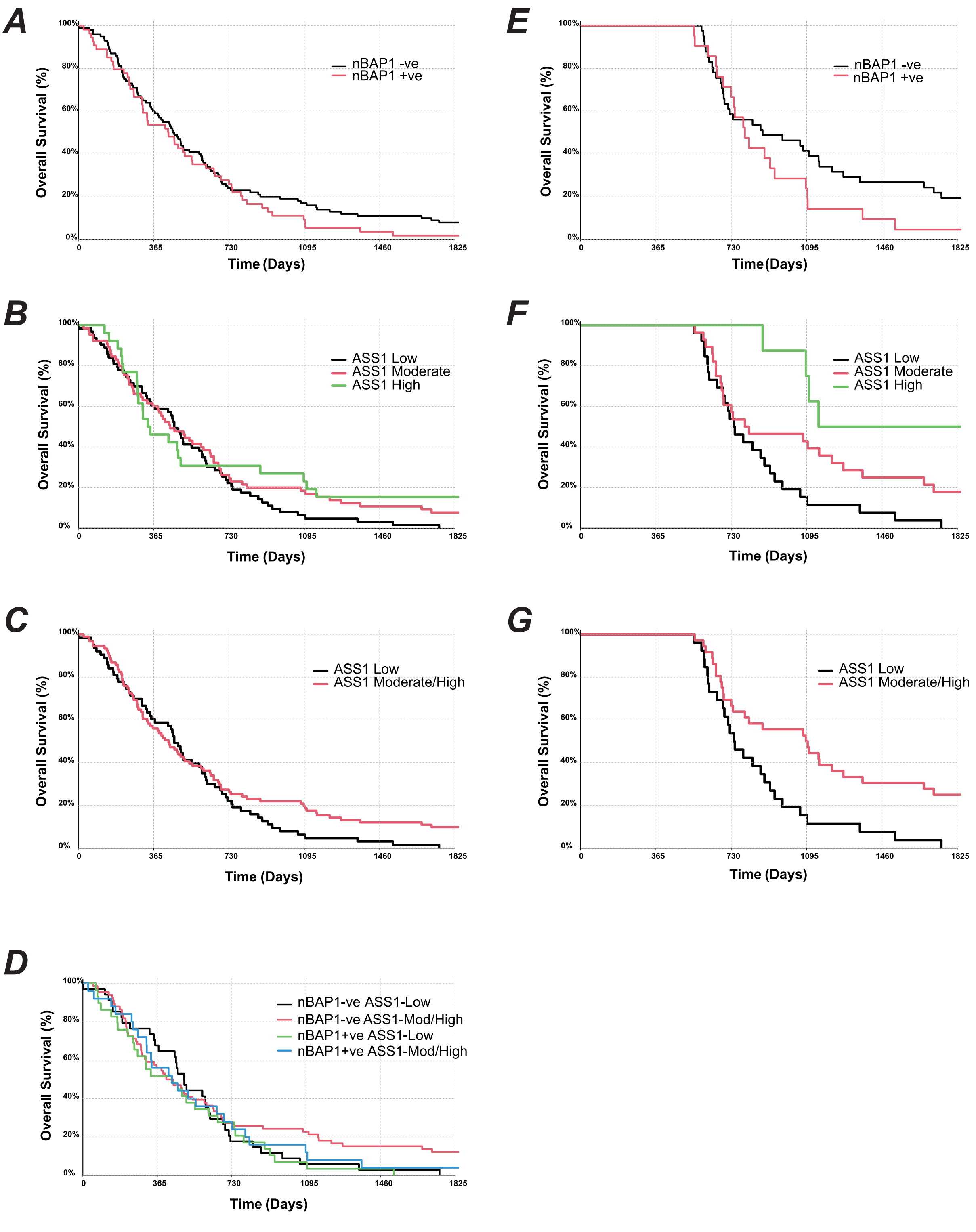

Supplementary Figure S13. Improved prognosis for epithelioid MPM patients with loss of nBAP1 and increased expression of ASS1.
A-D, Kaplan-Meier curves for all patients where overall survival data were available (n=154). **E-G,** Kaplan-Meier curves for landmark analysis at 18 months (n=62). Patient samples are stratified as nBAP1-negative versus nBAP1-positive (**A, E**); ASS1 H-score categorised as $ASS1_L$, $ASS1_M$ and $ASS1_H$ (**B, F**) or as $ASS1_L$ and $ASS1_{M/H}$ (**C, G**); and nBAP1-negative versus nBAP1-positive categorised by $ASS1_L$ and $ASS1_{M/H}$ (**D**). Supporting statistics for Cox models are in Supplementary Table S5.

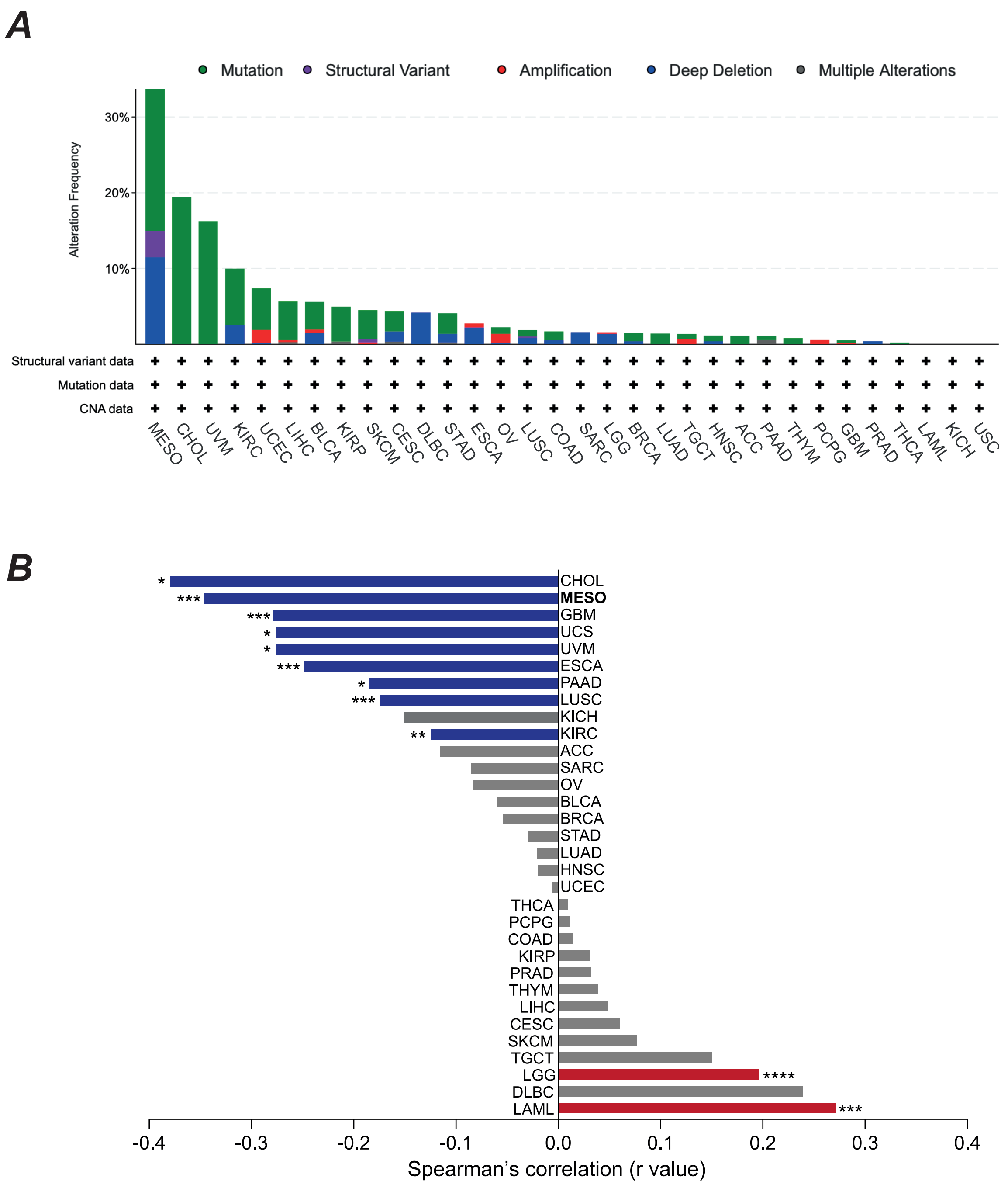

Supplementary Figure S14. Relationship between BAP1 and ASS1 transcripts in the TCGA Pan-Cancer datasets for other cancer types.
A, *BAP1* is most frequently altered in mesothelioma (MESO), cholangiocarcinoma (CHOL), uveal melanoma (UVM) and kidney renal clear cell carcinoma (KIRC). Summary of the frequency of *BAP1* genetic alteration within the TCGA pan-cancer data from 32 cancer types exported from cBioportal. The TCGA study abbreviations are available at https://gdc.cancer.gov/resources-tcga-users/tcga-code-tables/tcga-study-abbreviations. **B,** Inverse correlation of ASS1 and BAP1 transcripts is evident in the cancers where *BAP1* is most frequently mutated. Butterfly plot illustrating Spearman's correlation between ASS1 and BAP1 transcripts in each cancer type. Studies with significant inverse (blue) or positive (red) correlations are indicated; *P<0.05, **P=0.01, ***P=0.005, ****P=0.001. Results are based upon data generated by the TCGA Research Network: https://www.cancer.gov/tcga.

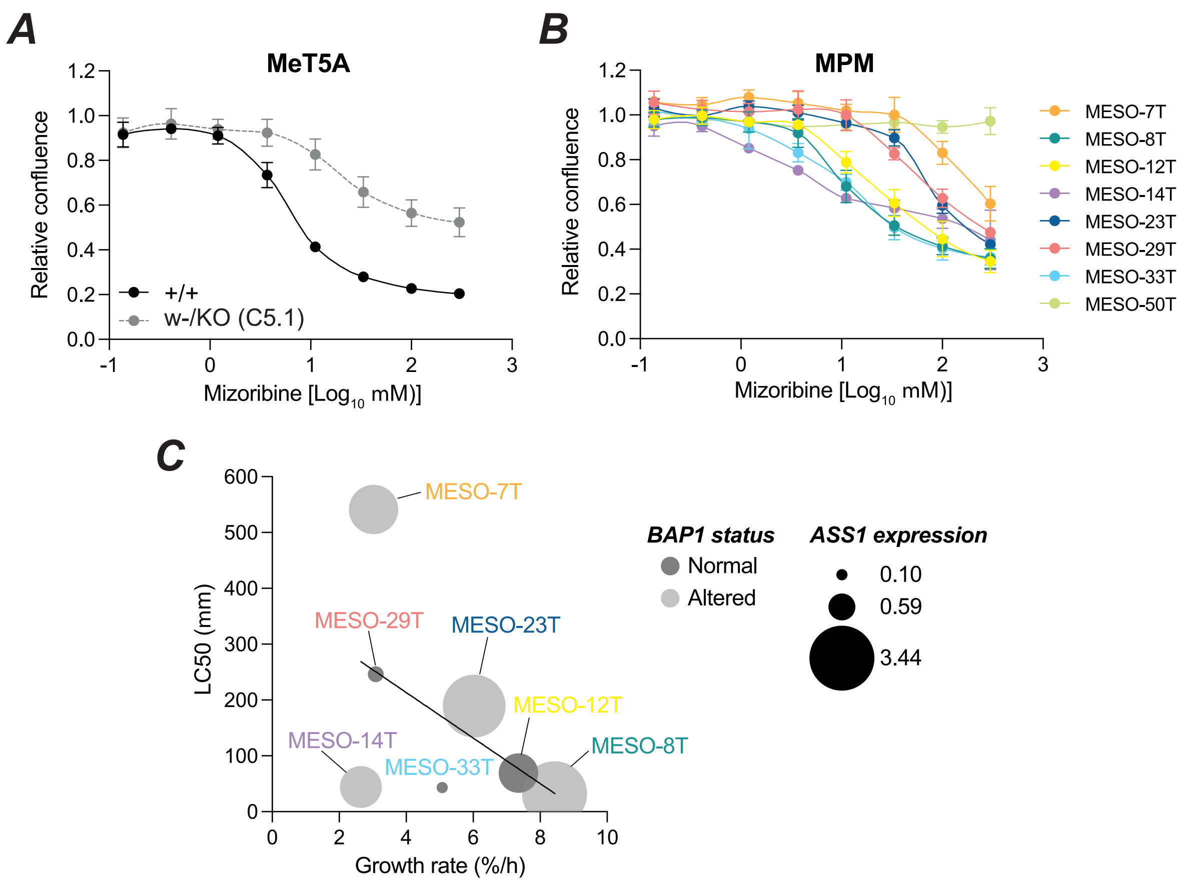

Supplementary Figure S15. The influence of BAP1-status on response to inhibition of purine metabolism.

A-C, Proliferative capacity rather than ASS1 or BAP1-status is more closely related to response to mizoribine. Relative confluence assessed by live imaging, mean of 3 independent experiments, error bars SD. Dose response curves for *BAP1*$^{+/+}$ and *BAP1*$^{w-/KO}$ MeT5A cells (**A**) or a panel of MPM cell lines (**B**) treated for 72h with mizoribine. Bubble plot shows the relationship between sensitivity to mizoribine and ASS1 level, BAP1-status, or growth rate (**C**).

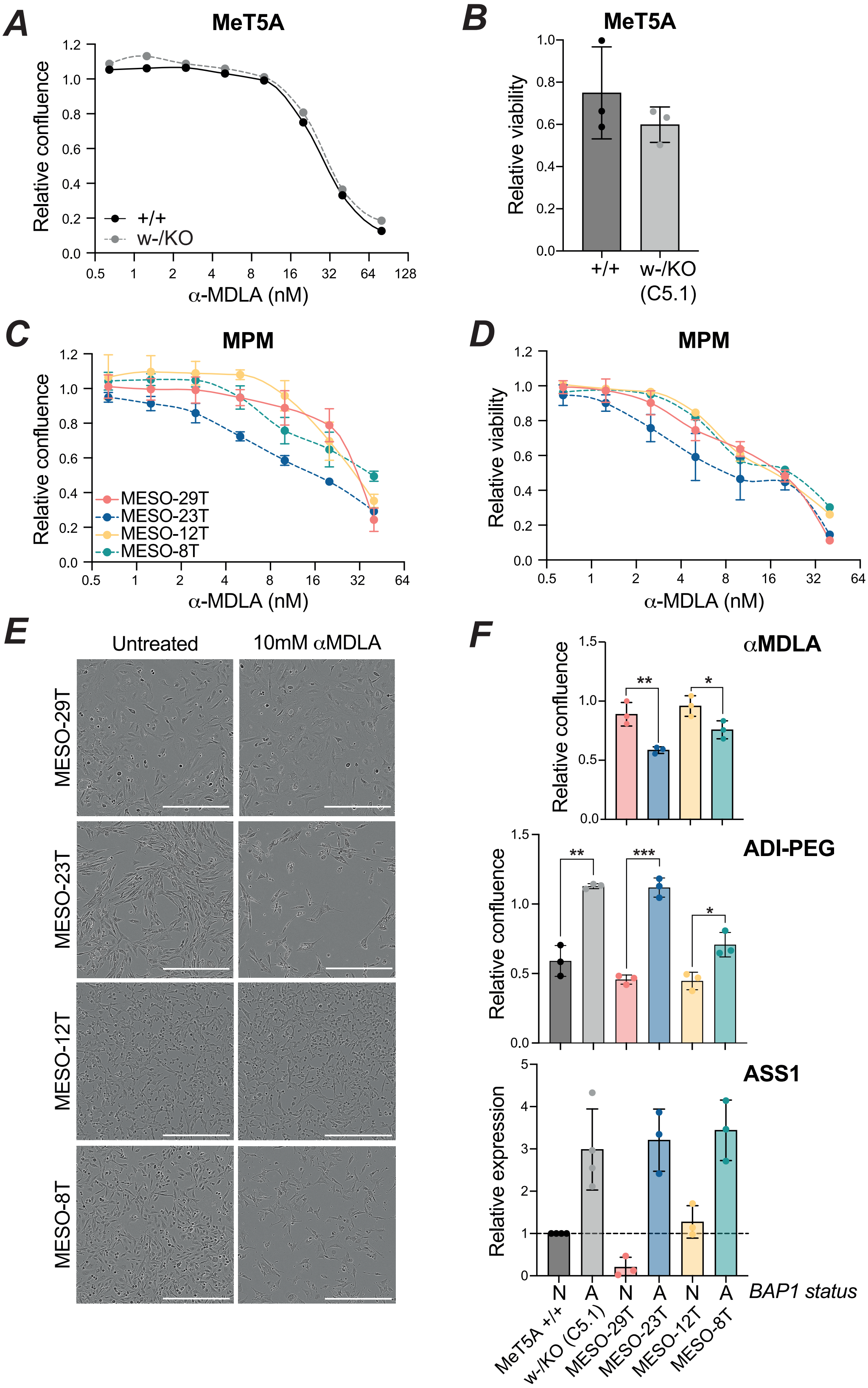

Supplementary Figure S16. The influence of BAP1-status on response to ASS1 inhibition.
A-B, MeT5A *BAP1*$^{+/+}$ and *BAP1*$^{w-/KO}$ cells were treated with the ASS1 inhibitor αMDLA. Dose response for relative confluence assessed by live imaging at 72h (**A**), n=1. Cell viability assessed by ATP-based luciferase assay at 72h for 10mM αMDLA (**B**), mean of 3 independent experiments, relative to vehicle control, error bars SD, unpaired t-test, P=0.327 (n.s.). **C-E**, Dose response for 72h αMDLA treatment in MPM cell lines: BAP1-normal, solid lines; BAP1-altered, dashed lines. Relative confluence assessed by live imaging (**C**) and relative viability assessed by ATP-based luciferase assay (**D**); mean of 3 independent experiments (2 for MESO-12T/8T viability), relative to vehicle control, error bars SD. Representative cell images at 72h for 10mM αMDLA (**E**). **F,** MPM cells exhibit inverse sensitivity to αMDLA and ADI-PEG20 that is related to ASS1 protein expression and BAP1-status. Bar charts show mean of 3 independent experiments, error bars SD, unpaired t-test. αMDLA: 10mM at 72h; **P*= 0.039, ***P*= 0.007. ADI-PEG20: 1000ng/mL at 72h; **P*= 0.014, ***P*= 0.0012, ****P*= 0.0001.
